# Biogeography of Bacterial Communities and Specialized Metabolism in Human Aerodigestive Tract Microbiomes

**DOI:** 10.1128/Spectrum.01669-21

**Published:** 2021-10-27

**Authors:** Reed M. Stubbendieck, Susan E. Zelasko, Nasia Safdar, Cameron R. Currie

**Affiliations:** a Department of Bacteriology, University of Wisconsin-Madison, Madison, Wisconsin, USA; b Microbiology Doctoral Training Program, University of Wisconsin-Madison, Madison, Wisconsin, USA; c Department of Medicine, University of Wisconsin-Madison, Madison, Wisconsin, USA; d Department of Medicine, University of Wisconsin-Madison School of Medicine and Public Health, Madison, Wisconsin, USA; e Department of Energy Great Lakes Bioenergy Research Center, University of Wisconsin-Madison, Madison, Wisconsin, USA; f Laboratory of Genetics, University of Wisconsin-Madison, Madison, Wisconsin, USA; Emory University School of Medicine

**Keywords:** *Actinomyces*, antibiotics, biosynthetic gene cluster, oral microbiome, nasal microbiome, natural products, RiPP, secondary metabolites, specialized metabolites, *Streptococcus*

## Abstract

The aerodigestive tract (ADT) is the primary portal through which pathogens and other invading microbes enter the body. As the direct interface with the environment, we hypothesize that the ADT microbiota possess biosynthetic gene clusters (BGCs) for antibiotics and other specialized metabolites to compete with both endogenous and exogenous microbes. From 1,214 bacterial genomes, representing 136 genera and 387 species that colonize the ADT, we identified 3,895 BGCs. To determine the distribution of BGCs and bacteria in different ADT sites, we aligned 1,424 metagenomes, from nine different ADT sites, onto the predicted BGCs. We show that alpha diversity varies across the ADT and that each site is associated with distinct bacterial communities and BGCs. We identify specific BGC families enriched in the buccal mucosa, external naris, gingiva, and tongue dorsum despite these sites harboring closely related bacteria. We reveal BGC enrichment patterns indicative of the ecology at each site. For instance, aryl polyene and resorcinol BGCs are enriched in the gingiva and tongue, which are colonized by many anaerobes. In addition, we find that streptococci colonizing the tongue and cheek possess different ribosomally synthesized and posttranslationally modified peptide BGCs. Finally, we highlight bacterial genera with BGCs but are underexplored for specialized metabolism and demonstrate the bioactivity of *Actinomyces* against other bacteria, including human pathogens. Together, our results demonstrate that specialized metabolism in the ADT is extensive and that by exploring these microbiomes further, we will better understand the ecology and biogeography of this system and identify new bioactive natural products.

**IMPORTANCE** Bacteria produce specialized metabolites to compete with other microbes. Though the biological activities of many specialized metabolites have been determined, our understanding of their ecology is limited, particularly within the human microbiome. As the aerodigestive tract (ADT) faces the external environment, bacteria colonizing this tract must compete both among themselves and with invading microbes, including human pathogens. We analyzed the genomes of ADT bacteria to identify biosynthetic gene clusters (BGCs) for specialized metabolites. We found that the majority of ADT BGCs are uncharacterized and the metabolites they encode are unknown. We mapped the distribution of BGCs across the ADT and determined that each site is associated with its own distinct bacterial community and BGCs. By further characterizing these BGCs, we will inform our understanding of ecology and biogeography across the ADT, and we may uncover new specialized metabolites, including antibiotics.

## INTRODUCTION

From the skin to the gastrointestinal (GI) tract, microbes colonize nearly every surface of the human body. The composition of these microbial communities varies across geographic scales, which can be as minute as different surfaces of the same tooth (1). This biogeography is controlled by abiotic factors, such as oxygen levels, pH, and salivary flow in the oral cavity (2, 3), and by biotic factors, which include competitive microbial interactions (4). Members of the endogenous microbiota engage in competition among themselves as well as with pathogens and other invading microbes (5). Together, these competitive interactions directly affect the population dynamics and spatial patterning of the microbiota across the human body.

Bacteria have evolved numerous systems to mediate competition, which include contact-dependent mechanisms, such as type VI secretion systems, and contact-independent mechanisms through the production of specialized metabolites (also called “secondary metabolites” and “natural products”) (6, 7). The complex competitive interactions that occur between microbes include exploitation or interference competition. In exploitation competition, one organism indirectly inhibits the growth of its competitors by preventing them from accessing resources, including nutrients and physical space. In interference competition, one organism produces toxic effectors that directly harm or inhibit its competitors. Microbes use specialized metabolites for both exploiting and interfering with the microbes they compete with for resources. For example, siderophores bind and sequester iron and other metals in exploitation, while antibiotics disrupt physiological processes in target cells as part of interference. It is important to note that these modes of competition are not mutually exclusive. Within the GI tracts of humans and other animals, colonization resistance against pathogens mediated by the endemic microbiota involves exploitation through niche occlusion (8) and interference, including type VI secretion systems (9) and production of metabolites (10).

In bacteria and other microbes, the genes responsible for production of specialized metabolites are organized in genomic loci called biosynthetic gene clusters (BGCs). These BGCs encode proteins necessary for biosynthesis, regulation, export, and self-resistance to specialized metabolites. Further, by characterizing the protein domains contained within synthetases and identifying the other biosynthetic enzymes encoded by a given BGC, one may classify the type of specialized metabolite it encodes (11). In some classes, in particular with nonribosomal peptide and type I polyketide BGCs, the core chemical structure encoded may be inferred by understanding the logic of assembly line enzymology (12). Bioinformatic approaches have been developed that enable the identification of putative BGCs from genomic and metagenomic sequences, leading to the identification of specialized metabolites in a genome-first manner. For example, the lactocillin BGC was first identified in metagenomic assemblies produced by the Human Microbiome Project before the antibiotic was purified from Lactobacillus gasseri cultures (13). The wexrubicin BGC was identified in GI tract metagenomes, amplified from Blautia wexlerae, and heterologously expressed to yield an anthracycline antibiotic (14). Further, by following the biosynthetic logic for nonribosomal peptides, total chemical synthesis was used to produce the antibiotic humimycins encoded by *Rhodococcus* species genomes (15). Together, these and other examples have led to a newfound appreciation of the biosynthetic potential of animal microbiomes for the identification of new bioactive natural products (16–20).

Previous efforts to characterize the specialized metabolite biosynthetic potential of human microbiomes have focused primarily on the GI tract (21, 22). Though the GI tract contains most of the microbial biomass of the human body and possesses a diverse bacterial community (23, 24), it does not directly receive input from the external environment. Instead, for exogenous microbes to reach the GI tract, they must first pass through the aerodigestive tract (ADT) (25). The ADT is a continuous multiorgan system that comprises the upper respiratory and digestive tracts, including the nasal and oral cavities (26). In contrast to the GI tract, the ADT directly faces the external environment. Therefore, in addition to competing among themselves, members of the ADT microbiota compete with exogenous microbes from the external environment, including human pathogens, and act as a barrier against colonization of the lower digestive and respiratory tracts by invaders (27, 28). Though many ADT sites possess a microbiota less diverse than that of the GI tract (23, 29, 30), bacterial genera found in the ADT, including *Corynebacterium*, *Staphylococcus*, and *Streptococcus* species, are known producers of specialized metabolites. For instance, bacteria that colonize the nasal cavity have previously been reported to produce the specialized metabolites dehydroxynocardamine (31), lugdunin (32), and nukacin IVK45 (33). Similarly, there is extensive literature on bacteriocin-mediated competition between oral bacteria, particularly within the context of dental caries (34, 35). These examples demonstrate the potential of the ADT microbiota to produce specialized metabolites, but our understanding of the natural ecology of these metabolites remains limited. By surveying BGCs from the ADT microbiota, we will better understand the capability of these bacteria to produce specialized metabolites, determine which genera to target for natural product discovery efforts, and inform our understanding of mechanisms underlying the biogeography of the upper digestive and respiratory tracts.

In the present study, we identified specialized metabolite BGCs in the genome sequences of bacteria that colonize the ADT and found extensive capacity for specialized metabolism among members of the ADT microbiota. By comparing these BGCs with experimentally characterized BGCs, we determined that most of the ADT BGCs are uncharacterized and the metabolites they encode are unknown. We then mapped metagenomic reads onto the ADT BGCs and used clustering analyses to identify distribution patterns of bacterial communities and specialized metabolism across the ADT. Using Shannon’s diversity index, we surveyed the alpha diversity of bacterial communities and BGCs across the ADT and determined that these diversity metrics are correlated. We then determined that the buccal mucosa, external naris, gingiva, and tongue dorsum are each enriched with specific families of BGCs, despite harboring many of the same bacterial genera. We found that the distribution of BGC families reflects potential ecological interactions that occur at and between the microbiota colonizing different ADT sites, suggestive of potential mechanisms that contribute to the biogeography of these sites. Finally, we performed targeted isolation of *Actinomyces*, a genus that has been underexplored for specialized metabolism, from the oral cavity and demonstrated their bioactivity against a panel of bacteria, including human pathogens. These data provide new insights into the potential for specialized metabolism among the ADT microbiota, inform our understanding of the ecology of this system, and highlight bacterial genera that have been underappreciated for natural product discovery.

## RESULTS AND DISCUSSION

### ADT bacteria possess BGCs for specialized metabolism.

Bacteria that colonize the ADT produce antibiotics and other specialized metabolites to compete with other members of the native microbiota and invading microbes from the external environment. In this study, we characterized the potential for specialized metabolism of ADT microbiomes by identifying BGCs from the ADT bacteria and surveying how these BGCs are distributed across different sites in this multiorgan system.

**FIG 1:**
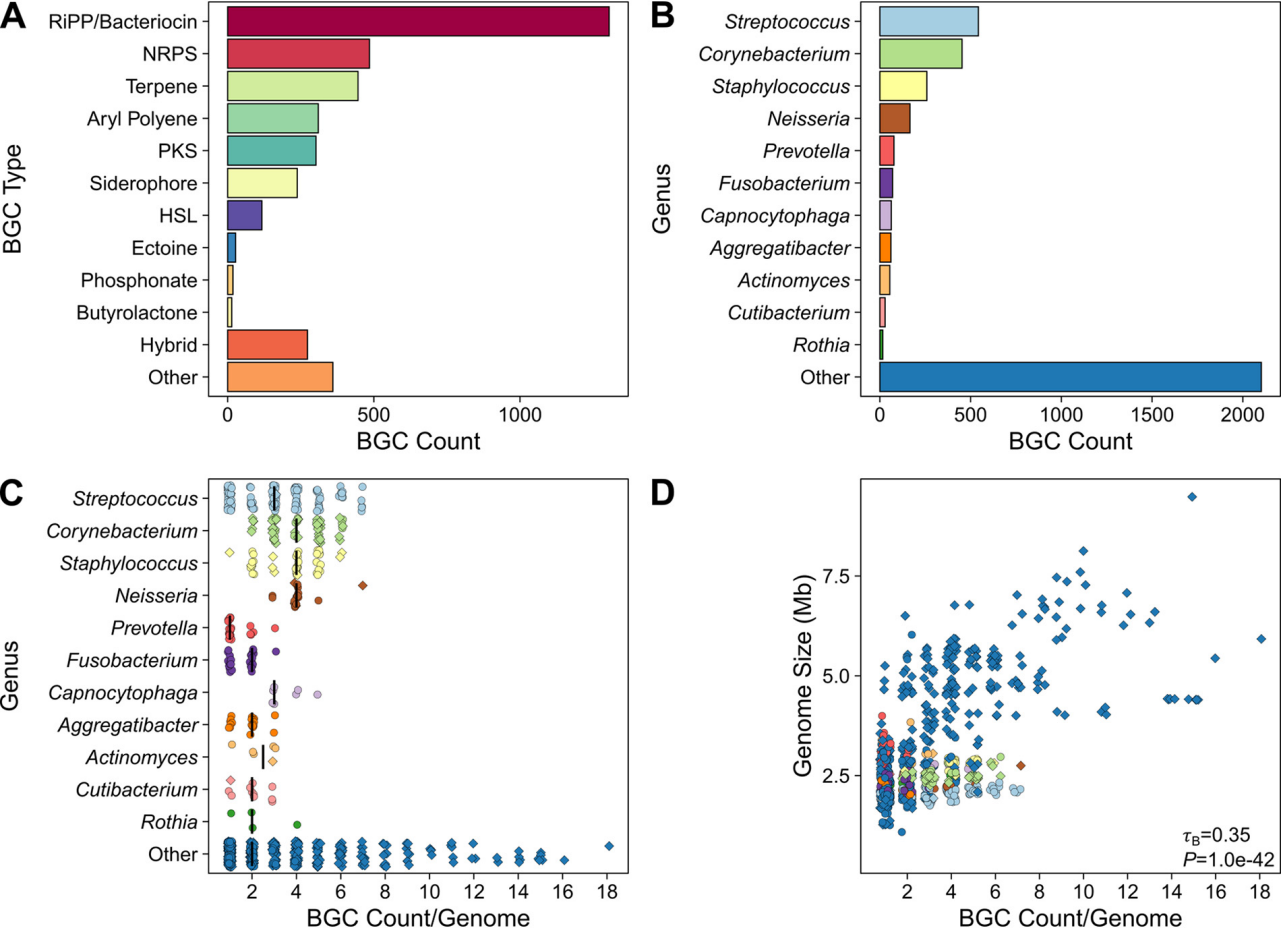
Human ADT bacteria possess gene clusters for specialized metabolism. (A) Bar chart indicating the total count of BGCs in the eHOMD genomes separated by BGC type. (B) Bar chart indicating the total counts of BGCs in the eHOMD genomes by bacterial genus. (C) Plot indicating the number of BGCs per genome for common ADT bacterial genera. Each point represents a genome from a single strain of the corresponding genus. The vertical black bars represent the median BGC count per genome. (D) Plot indicating the relationship between eHOMD genome size and BGC count. Each point represents a genome from a single strain and is colored by genus as in panel C. The Kendall rank correlation coefficient (τ_B_) and *P* value between genome size and BGC count is shown in the lower right corner of the panel. Genomes that did not contain BGCs or were <1 Mb in total length were excluded from these analyses. In panels C and D, the shape of the points indicates the source of the strains, with circles representing the genomes of bacteria known to colonize the ADT (eHOMD habitat listed as “Nasal,” “Nasal,Oral,” or “Oral”) and diamonds representing the genomes of environmental bacteria that are transiently found in the ADT (eHOMD habitat listed as “NonOralRef,” “Skin,” “Unassigned,” or “Vaginal”). The points in panels C and D were jittered to avoid overplotting. The data in panels A and B include fragmented BGCs located on contig edges, whereas the data in panels C and D include only genomes where no fragmented BGCs were detected.

Using antiSMASH (36), we searched for putative BGCs from the expanded human oral microbiome database (eHOMD), which contains genome sequences from bacteria that colonize different sites across the ADT and reference genomes for environmental bacteria that may be found transiently within the ADT (30). We identified BGCs from 1,214 genomes, representing 136 genera and 387 species. In total, we predicted 3,162 full-length BGCs and 733 BGC fragments located on contig edges, including ribosomally synthesized and posttranslationally modified peptide (RiPP) and bacteriocin BGCs (together referred to as RiPP/bacteriocin BGCs), nonribosomal peptide synthase (NRPS) BGCs, terpene BGCs, aryl polyene BGCs, polyketide synthase (PKS) BGCs, *iucA*/*iucC*-containing siderophore BGCs (referred to as siderophore BGCs for simplicity), homoserine lactone (HSL) BGCs, phosphonate BGCs, and butyrolactone BGCs (Fig. 1A; Table 1; Table S1). In addition to these BGCs, we also predicted hybrid BGCs, consisting of two or more BGCs located adjacent to each other within the genome (Fig. 1A; Table 1; Table S1). A total of 58% of the full-length NRPS BGCs contained only two adenylation domains (Fig. S1) and may encode the biosynthesis of diketopiperazines (37). Among the full-length type I PKS BGCs we predicted, 61% were predicted from *Corynebacterium* or *Mycobacterium* genomes and contained a single ketosynthase domain, suggesting that these “clusters” actually encode mycolic acid condensase and are not responsible for specialized metabolite biosynthesis (38) (Fig. S1). Previously, Aleti et al. identified 5,611 total clusters from 461 genomes, representing 113 bacterial genera and 298 species, using the Human Oral Microbiome Database (39). After removing fatty acid clusters, saccharide clusters, and putative clusters of unknown type, their study identified 853 BGCs (Table 1). Direct comparison in the total number of identified BGCs between these two studies is not recommended, as Aleti et al. chose to examine one genome per bacterial species (39), whereas we included all available genomes in the eHOMD to maximize the diversity of observed BGCs. However, we identified an overall composition of BGCs from ADT and environmental bacteria similar to that found by Aleti et al. found in their study (Table 1).

**TABLE 1:** BGCs identified in genomes of ADT and environmental bacteria from the eHOMD^*a*^

BGC type	Count from ADT bacteria	Count from environmental bacteria	Total count from ADT and environmental bacteria (%)	Count (%) from Aleti et al. (39)
Bacteriocin	610	252	862 (22.1%)	67 (7.9%)
RiPP^*b*^	241	202	443 (11.4%)	151 (17.7%)
NRPS	129	356	485 (12.5%)	101 (11.8%)
Terpene	150	296	446 (11.5%)	98 (11.5%)
Aryl Polyene	211	99	310 (8.0%)	147 (17.2%)
PKS^*c*^	105	197	302 (7.8%)	85 (10.0%)
Siderophore	100	138	238 (6.1%)	28 (3.3%)
HSL	11	106	117 (3.0%)	35 (4.1%)
Ectoine	0	27	27 (0.7%)	8 (0.9%)
Phosphonate	0	18	18 (0.5%)	7 (0.8%)
Butyrolactone	9	5	14 (0.4%)	6 (0.7%)
Hybrid^*d*^	60	213	273 (7.0%)	54 (6.3%)
Other^*e*^	106	254	360 (9.2%)	66 (7.7%)
Total	1,732	2,163	3,895 (100%)	853 (100%)

a ADT indicates bacteria that are known colonizers of the ADT (eHOMD habitat listed as “Nasal,” “Nasal,Oral,” or “Oral”), and environment indicates environmental bacteria that are transiently found in the ADT (eHOMD habitat listed as “NonOralRef,” “Skin,” “Unassigned,” or “Vaginal”).

b The RiPP category contains cyanobactin, glycocin, lantipeptide, lassopeptide, linaridin, microcin, proteusin, sactipeptide, and thiopeptide BGCs.

c The PKS category contains other ketosynthase, resorcinol, type I PKS, type II PKS, type III PKS, and transAT-PKS BGCs.

d Hybrid indicates two or more BGCs located adjacent to each other within the genome.

e The other category contains acyl amino acid BGCs, beta-lactam BGCs, ladderane BGCs, phenazine BGCs, and BGCs that encode a protein involved in specialized metabolism but do not fit into any currently known biosynthetic category.

In decreasing order of total BGC count, *Streptococcus*, *Corynebacterium*, *Staphylococcus*, *Neisseria*, *Prevotella*, *Fusobacterium*, *Capnocytophaga*, *Aggregatibacter*, *Actinomyces*, *Cutibacterium*, and *Rothia* genomes contained 1,793 BGCs (46%) (Fig. 1B). In addition, there was a long tail consisting of 125 other genera with 2,102 BGCs (54%) (Fig. 1B). Similarly, 1,732 BGCs (44%) were predicted from the genomes of ADT colonizers and 2,163 BGCs (56%) were predicted from the genomes of environmental bacteria. Within genomes of both ADT and environmental bacteria containing at least one BGC and with no fragmented BGCs, the total number of BGCs per genome ranged from 1 to 18, with a median value of 2 (interquartile range [IQR]: 1 to 4 BGCs per genome) (Fig. 1C). The genomes of ADT bacteria contained 1 to 7 BGCs per genome with a median value of 2 (IQR: 1 to 3 BGCs per genome). Overall, the BGC count per genome was positively correlated with genome size (Fig. 1D), which is consistent with a previous global analysis of BGCs within bacterial genomes (40). Together, these genomic analyses indicate that bacteria colonizing the ADT possess vast potential for specialized metabolism and should be further investigated for the identification of bioactive natural products.

### Each ADT site harbors a distinct microbiota and suite of BGCs.

**FIG 2:**
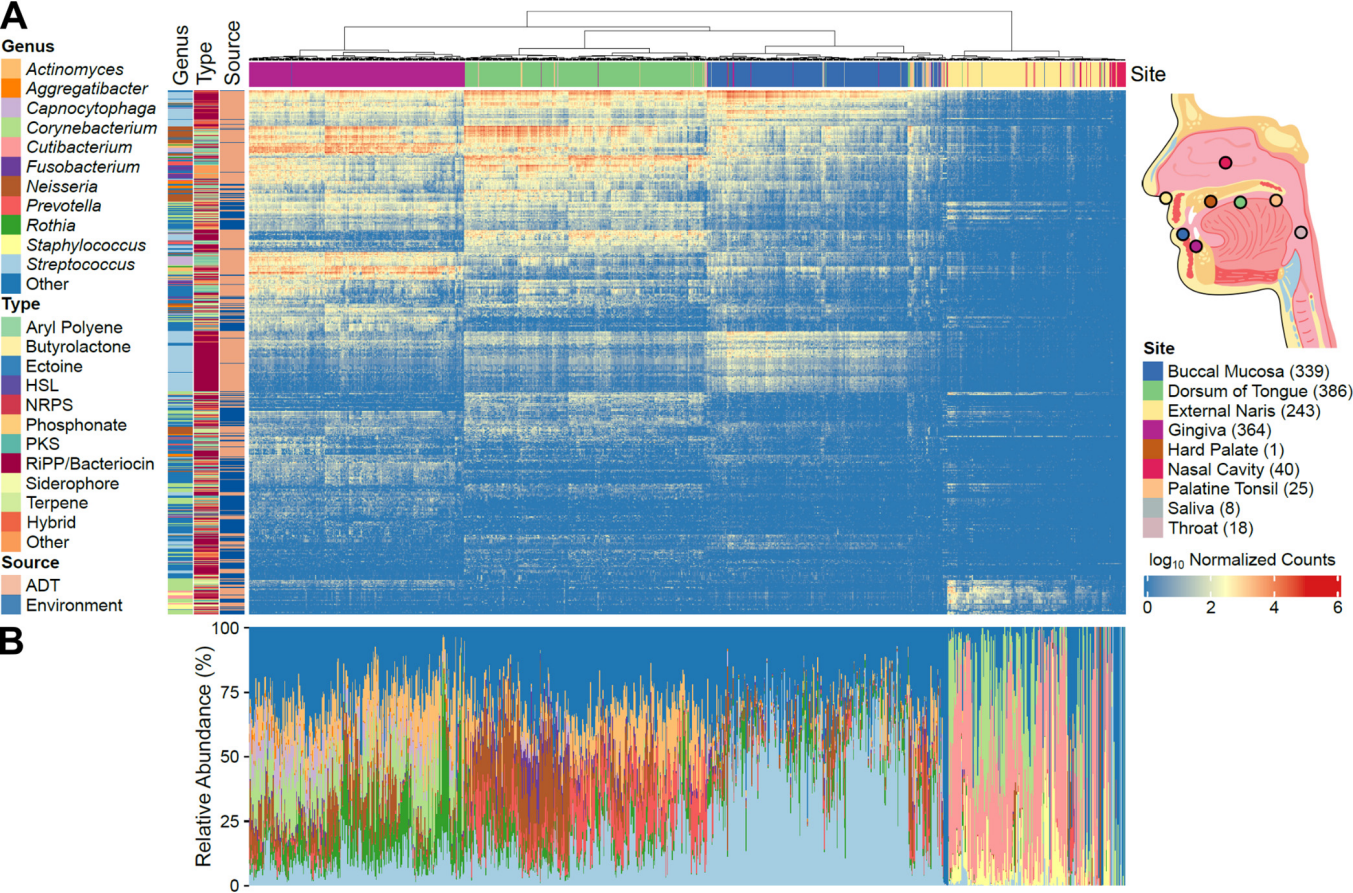
The composition of specialized metabolite BGCs in ADT microbiomes cluster based on site. (A) Metagenomic reads from 1,424 samples (columns) were processed and pseudoaligned onto 2,094 BGCs (rows). The heat map displays the size factor-normalized counts of reads that mapped to each BGC in each sample. BGCs with <100 total normalized reads aligned across all metagenomes were filtered from this heat map. The colored bar at the top of the heat map indicates the body site where the corresponding metagenomic sample was derived. The inset on the right indicates the relative positions of these sites on a sagittal cross section of the human ADT. The palatine tonsil is behind the tongue in this view and saliva is not depicted. The total number of metagenome samples from each ADT site is indicated within parentheses in the key. The colored bars on the left of the heat map indicate the predicted BGC type from antiSMASH, the bacterial genus from which the corresponding BGC was identified, and the source of the strain. ADT indicates bacteria that are known colonizers of the ADT (eHOMD habitat listed as “Nasal,” “Nasal,Oral,” or “Oral”) and Environment indicates environmental bacteria that are transiently found in the ADT (eHOMD habitat listed as “NonOralRef,” “Skin,” “Unassigned,” or “Vaginal”). The columns and rows of the heat map were clustered hierarchically. For clarity, only the clustering dendrogram for the metagenome sites is shown. (B) Stacked bars indicate the relative abundance of different bacterial genera within the corresponding metagenomes in panel A. Inset diagram was illustrated by Julia Buskirk (University of Wisconsin-Madison).

As bacterial communities differ across the ADT (23), we hypothesized that the microbiota associated with each ADT site may possess its own characteristic set of BGCs. To identify BGCs associated with different sites along the ADT, we mapped metagenomic reads from 1,424 ADT samples collected by the Integrative Human Microbiome Project (iHMP) (41) (Table S2) onto the set of BGCs that we predicted above (Fig. 2A). These ADT metagenome samples were collected from the buccal mucosa (the inner lining of the cheeks and back of the lips), dorsum of the tongue, external naris, gingiva, hard palate, nasal cavity, palatine tonsil, saliva, and throat (see Fig. 2, right for relative sampling locations). We found that significantly more reads mapped to BGCs identified from ADT colonizers (median: 2.3e05 estimated reads per sample [IQR: 4.0e04 to 5.0e05 estimated reads per sample]) than to BGCs from reference environmental bacteria (median: 2.0e04 estimated reads per sample [IQR: 4.9e03 to 4.1e04 estimated reads per sample]) [sign test, *S*(1,424) = 1,393, *P *< 1e−08] (Fig. S2A). Across all ADT metagenomes analyzed, the median percentage of reads mapping to BGCs from environmental bacteria was 9% (IQR: 6 to 14%) (Fig. S2B). The group of environmental bacteria in the eHOMD contains 24 genera that are also found in the group of known ADT colonizers (Fig. 1C; Table S1). Thus, reads from genuine ADT bacteria may map to BGCs that were predicted from environmental bacteria. Indeed, 41% of all reads mapping to *Corynebacterium* BGCs in the external naris mapped to BGCs identified from environmental reference genomes. Among these reference *Corynebacterium* were species such as Corynebacterium simulans and Corynebacterium tuberculostearicum, which are known members of the human nasal microbiome (42, 43). Therefore, given the above findings, we decided to consider each metagenome as a whole for all following analyses, instead of filtering out reads that mapped to the BGCs from environmental bacteria. The genera most highly enriched in BGCs were common ADT colonizers, including *Actinomyces*, *Aggregatibacter*, *Capnocytophaga*, *Corynebacterium*, *Fusobacterium*, *Neisseria*, *Prevotella*, and *Streptococcus*. In addition to focusing on these eight genera for subsequent analyses, we also included *Cutibacterium* and *Staphylococcus*, as common members of external naris and nasal cavity microbiomes (44), and *Rothia*, which is a frequent member of oral microbiomes (45) (Fig. 2B).

**FIG 3:**
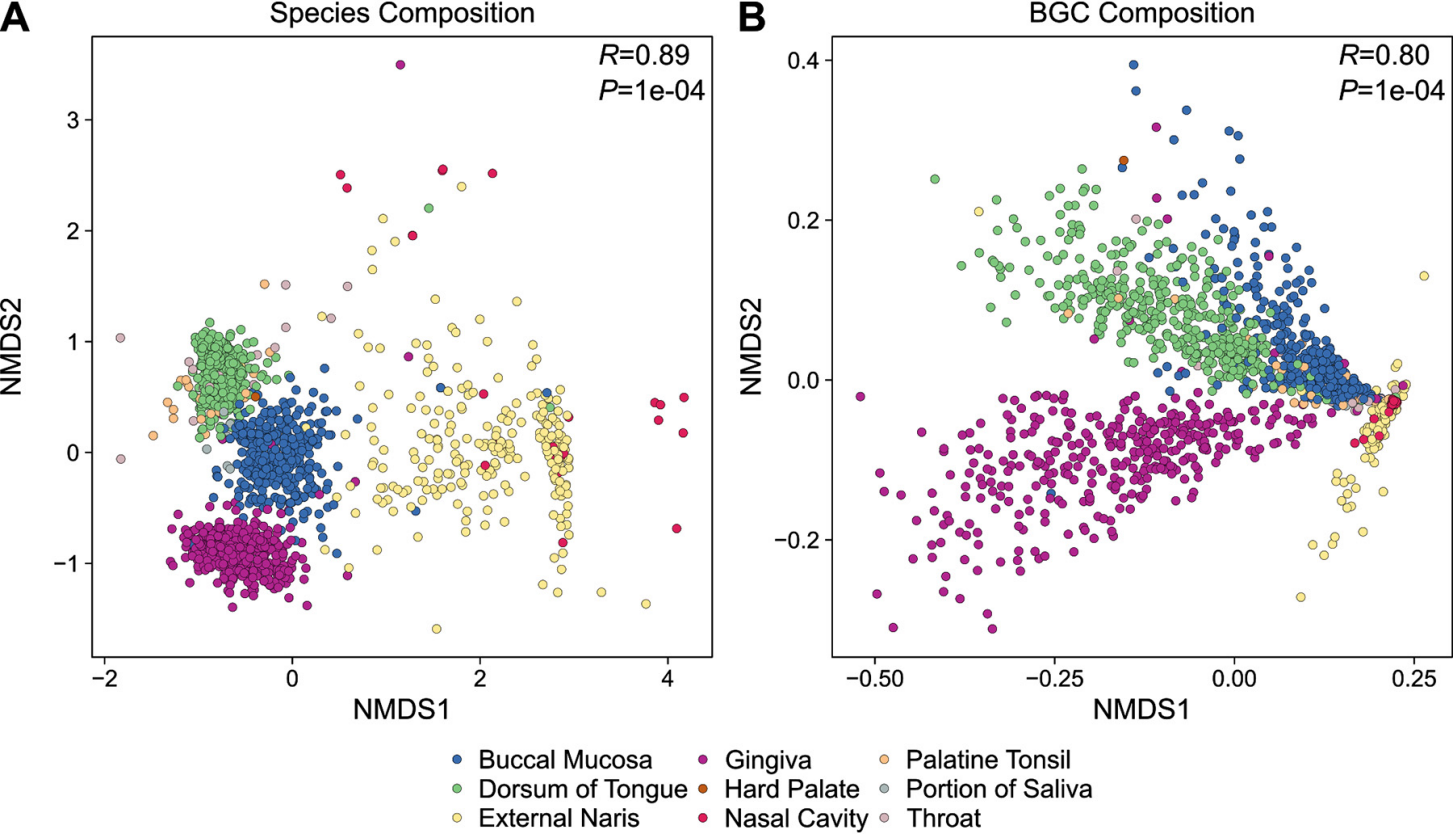
Bacterial species and BGC composition vary across the ADT. Nonmetric multidimensional scaling (NMDS) plots for (A) bacterial species and (B) BGCs detected in ADT metagenomes. Each point represents a single metagenome sample and is colored based on ADT site, as indicated in the key. The ANOSIM *R* and *P* values (10,000 permutations) are shown in the top right of each plot. The stress value for each NMDS plot was 0.11.

Using a hierarchical clustering analysis, we found that the BGC composition of ADT metagenomes was stratified primarily based on the corresponding body site, with the gingiva, tongue dorsum, buccal mucosa, and external naris and nasal cavity, together, each forming their own distinct clusters that were 85.7 to 99.7% pure (i.e., containing only metagenome samples from a single ADT site) (Fig. 2A, top). At the highest level, the BGC composition of the external naris and nasal cavity was dissimilar from sites in the oral cavity, likely reflecting broad differences in the bacteria and differences between members of shared taxa that colonize each site (Fig. 2B). Further, within the oral cavity, the BGC composition of the buccal mucosa and that of the tongue dorsum were more similar to each other than to that of the gingiva, but were also clearly separate from each other. We then used hierarchical clustering to analyze ADT bacterial communities at the species level. Similarly to BGCs, there was separation of the communities based on ADT site. Moreover, the hierarchical clusters of BGCs and bacterial communities agreed with each other (Jaccard similarity of 0.87). Subsequently, we confirmed that ADT site influences the composition of both bacterial community at the species level (analysis of similarity [ANOSIM] *R *= 0.89) (Fig. 3A) and BGCs (ANOSIM *R *= 0.80) (Fig. 3B). In summary, these data are consistent with our hypothesis and provide additional evidence for biogeography in the ADT, with respect to both bacterial communities and specialized metabolism.

### Bacterial community diversity and BGC diversity vary between and correlate within ADT sites.

**FIG 4:**
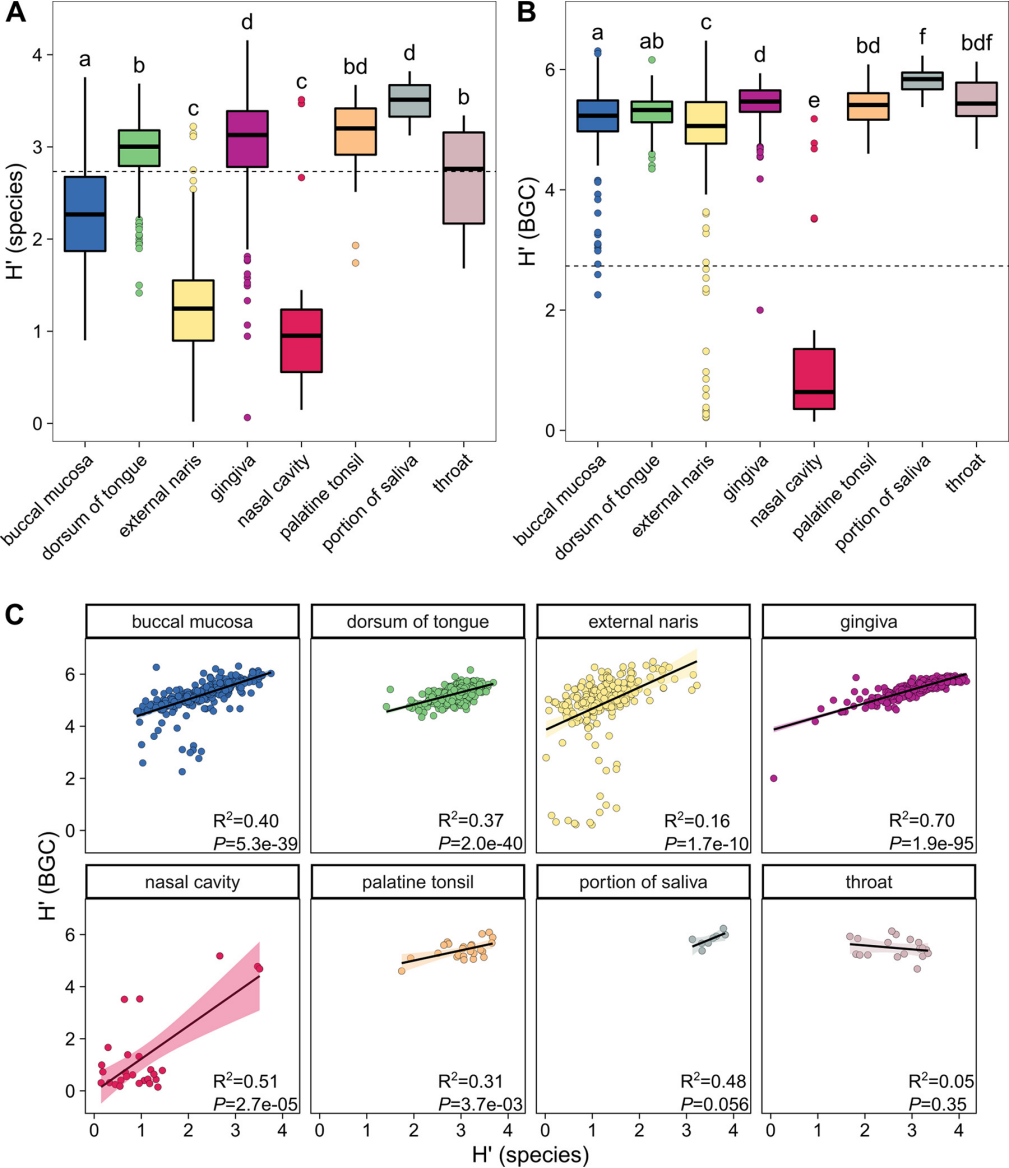
Bacterial species and BGC diversity are correlated across the ADT. Box plots of the Shannon diversity index (H′) for (A) species-level bacterial community and (B) BGC composition in the ADT. The upper and lower bounds of the box plots indicate the 75th and 25th percentiles, respectively. The horizontal black bars indicate the medians. The whiskers extend from the bounds of the box to the largest and smallest values that are no further than ±1.5× the IQR. All outliers that occur outside this range are shown as points. ADT sites that share letters are not significantly different (*α* = 0.05). The horizontal dashed lines indicate the overall median alpha diversity value for each panel. (C) Plots showing the relationship between H′ at the species and BGC levels. The black lines and colored shading represent the line of best fit and 95% confidence interval, respectively. The correlation coefficients (R^2^) and *P* values for linear models are shown in the lower right of each facet. For these analyses, samples with H′_species_ of 0 were removed.

We determined that the alpha diversity, estimated with the Shannon diversity index (H′), of bacterial communities differed throughout the ADT. In particular, saliva and the gingiva were colonized by the most diverse bacterial communities, while the external naris and nasal cavity maintained the least diverse communities (Fig. 4A). These findings are similar to those obtained in other surveys comparing the overall diversity of human nasal and oral microbiomes (26). However, we observed a higher relative alpha diversity of bacterial species in the gingiva from whole-genome shotgun metagenomics than previously reported using operational taxonomic units, based on amplicon sequencing of the 16S rRNA gene V3 to V5 region (23, 46). Similarly, we applied the H′ to assess the alpha diversity of BGCs and showed variation among different ADT sites. As with bacterial communities, saliva possessed the highest diversity of BGCs, while the nasal cavity was the least diverse ADT site (Fig. 4B). ADT site had a strong effect on community diversity (ε^2 ^= 0.57) and a more moderate effect on BGC diversity (ε^2^ = 0.15). Further, the alpha diversity of BGCs was higher than that of bacterial communities [sign test, *S*(1,407) = 1,384, *P *< 1e−08], which is consistent with multiple BGCs per bacterial genome (Fig. 1C).

We detected the highest number of bacterial species (i.e., richness [S]) in the oral cavity, particularly within the gingiva (median S: 112), palatine tonsil (median S: 91), saliva (median S: 123), and tongue dorsum (median S: 112) (Fig. S3A). Similarly, the gingiva contained the most distinct BGCs (median S: 1,883), followed by the tongue dorsum (median S: 1,681). BGC richness in saliva (median S: 1,679) was not distinguishable from either of these sites or from the buccal mucosa (median S: 1,458) and palatine tonsil (median S: 1,531) (Fig. S3B). With respect to both species and BGC richness, the external naris (species median S: 11, BGC median S: 692) and nasal cavity (species median S: 4, BGC median S: 302) possessed the lowest values among the ADT sites we analyzed (Fig. S3A and B). Using Shannon’s equitability index (E_H_), we determined that the distribution of bacterial species (median E_H_: 0.62 [IQR: 0.53 to 0.68]) (Fig. S3C) and BGCs (median E_H_: 0.73 [IQR: 0.70 to 0.75]) (Fig. S3D) in the ADT was relatively even and comparable to values obtained from a meta-analysis of species within the GI tract (overall E_H_: 0.66) (47). However, notably, BGCs in the nasal cavity were distributed less evenly (median E_H_: 0.12) than all other ADT sites, suggesting that these metagenomes are dominated by a small number of gene clusters. Indeed, a single uncharacterized aryl polyene BGC from Prevotella micans was highly abundant in 29/40 nasal cavity metagenomes in our study.

As many bacterial genomes contain their own repertoire of BGCs, we expected to observe correlations between community and BGC diversity. Indeed, we observed a significant positive correlation between species-level diversity and BGC diversity for all ADT sites overall (R^2 ^= 0.29, *P *= 1.2e−105). This correlation held for the buccal mucosa, tongue dorsum, gingiva, nasal cavity, and palatine tonsil (Fig. 4C). However, there was no significant relationship between the two Shannon diversity indices for saliva or the throat (Fig. 4C).

### Identification of BGCs and BGC families enriched in specific ADT microbiomes.

**FIG 5:**
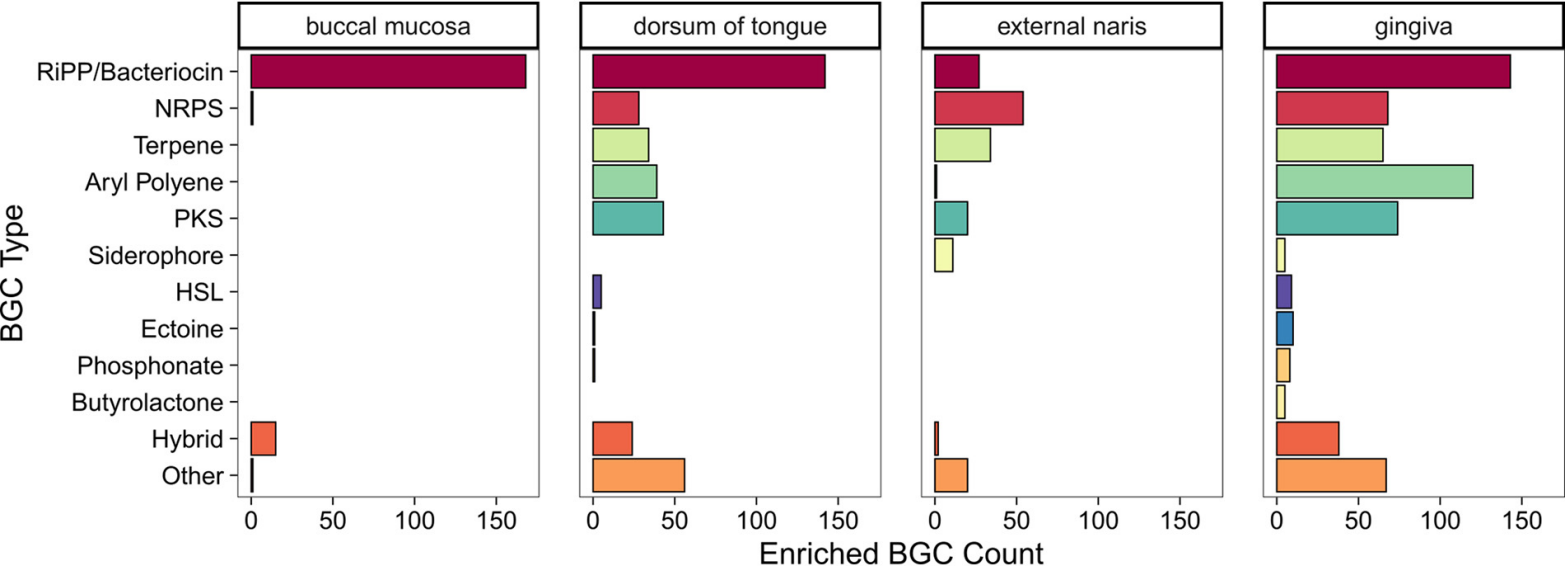
Enrichment of BGCs in ADT microbiomes. Bar chart indicating the total count of enriched BGCs in the buccal mucosa, dorsum of tongue, external naris, and gingiva separated by BGC type.

Given that the bacterial community and BGC composition of each ADT site was distinct (Fig. 2 and 3), we reasoned that each microbiome would be enriched with a particular set of BGCs that correlate with the specific bacteria that colonize these sites. To identify these BGCs, we performed a differential abundance analysis comparing BGC enrichment in a focal site over the other sites. For this and all following analyses, we chose to focus on the buccal mucosa, tongue dorsum, external naris, and gingiva, as these four sites were each represented by >200 metagenome samples, while the other sites were represented by <50 samples each. Of the 3,601 BGCs detected in at least one metagenome, 185 BGCs (Fig. S4A) were enriched in the buccal mucosa, 373 BGCs were enriched in the tongue dorsum (Fig. S4B), 169 BGCs were enriched in the external naris (Fig. S4C), and 613 BGCs were enriched in the gingiva (Fig. S4D). Thus, the enrichment analysis indicates that specific BGCs are associated with each ADT site (Fig. 5).

**FIG 6:**
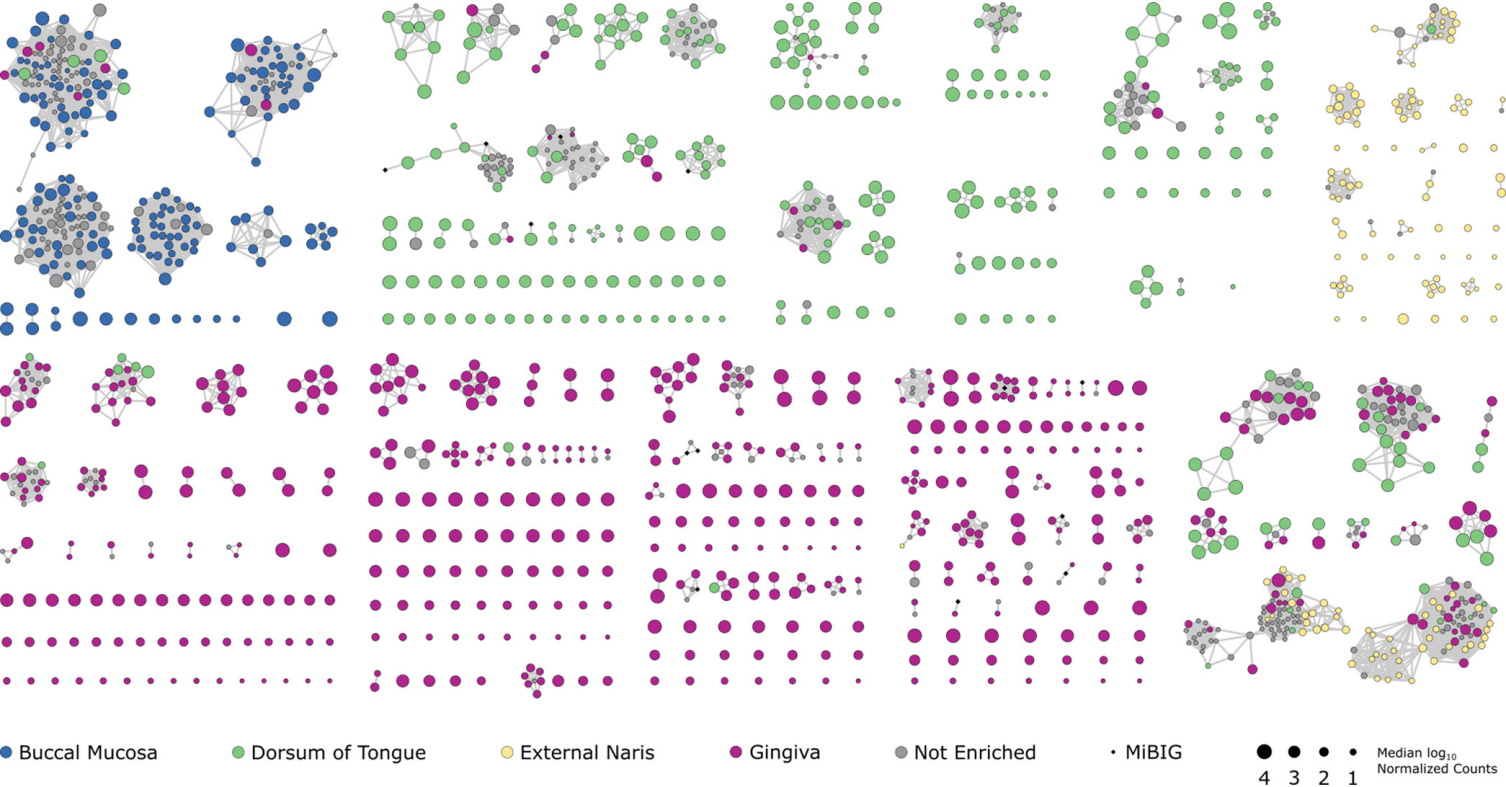
Identification of BGC families associated with specific ADT microbiomes. In this network, each node represents a single BGC and edges indicate weighted pairwise distances between the corresponding BGCs. The nodes are colored based on ADT site where that BGC was enriched, and their size is scaled based on the median log_10_ normalized count value of the BGC in that site (see black circles in the bottom for key). For BGCs that were not enriched in any site, the corresponding node sizes are scaled relative to the median value across all samples and are colored gray. Black diamonds represent characterized BGCs from the MIBiG database 1.4. See Figure S3A and B for the same network but with nodes colored by genus and BGC type, respectively. For clarity, BGC families with few or no enriched clusters were removed from this view.

Our finding that each ADT site was associated with a distinct group of BGCs prompted us to investigate the relationships among these BGCs and to ask if specific BGC families were associated with each ADT site. To assess relationships between ADT BGCs, we generated a BGC similarity network using BiG-SCAPE (48) annotated using information from our metagenomic read alignments (Fig. 2) and differential abundance analysis (Fig. 5). In addition to building the network from ADT BGCs, we included BGCs from the Minimum Information about a Biosynthetic Gene Cluster (MIBiG) database (49) to aid in identification of characterized BGCs. The total BGC network contained 3,972 nodes, representing 3,891 ADT BGCs and 81 MIBiG database BGCs, and 26,365 edges, each indicating a relationship between two BGCs (Fig. 6; Fig. S5). We highlight specific findings from this analysis below.

### Aryl polyene and resorcinol BGCs are enriched in the tongue dorsum and gingiva.

As a proof of principal that BGCs reflect potential ecological and physiological functions in ADT bacteria, we first examined BGCs that encode the biosynthesis of aryl polyenes and resorcinols. Aryl polyenes are widespread specialized metabolites synthesized by type II PKS that consist of a polyene carboxylic acid conjugated to a terminal aryl group (40, 50). Reactive oxygen species are generated in the oral cavity both through the action of microbes, in particular by mitis group streptococci (51), and by host inflammatory processes (52). To tolerate oxidative damage, bacteria use multiple strategies, including production of carotenoids and other antioxidants (53). Aryl polyenes can function analogously to carotenoids, acting as membrane-associated pigments that protect against both photo and oxidative damage by delocalizing unpaired electrons through the polyene moiety (54, 55). Obligate and facultative anaerobic bacteria, including *Actinomyces*, *Capnocytophaga*, and *Prevotella* species, are common colonizers of the human oral cavity (56, 57). In the gingiva and tongue dorsum, 120 and 39 aryl polyene BGCs, respectively, were enriched from anaerobic and facultative anerobic bacteria, including *Actinomyces*, *Capnocytophaga*, *Eikenella*, *Kingella*, *Prevotella*, *Tannerella*, and *Treponema* (Fig. S6; Table S1). Further, these BGCs were also enriched in the same sites from the microaerophilic bacterium Campylobacter and the aerobic bacterium *Neisseria* (Fig. S6; Table S1). Though no functional role has been demonstrated for any aryl polyene produced by a human oral bacterial strain, mutants of the environmental bacterium Variovorax paradoxus that were unable to produce aryl polyenes were more susceptible to oxidative damage than the wild-type strain (54).

Similarly to aryl polyenes, 38 and 26 resorcinol BGCs were enriched in the gingiva and tongue dorsum, respectively. These BGCs were from *Capnocytophaga*, *Porphyromonas*, and *Prevotella* species (Fig. S6; Table S1). Alkylresorcinols are phenolic lipids that contain a resorcinol head synthesized by type III PKS and conjugated to an unsaturated alkyl chain (58). These lipid metabolites possess antioxidant activity and have been shown to protect Escherichia coli from superoxide stress (59, 60). Thus, the enrichment of aryl polyene and resorcinol BGCs in the oral cavity suggests that these metabolites may contribute to protection against oxidative damage *in situ* (40).

### Oral streptococci that colonize the tongue and cheek harbor different RiPP-encoding BGCs.

**FIG 7:**
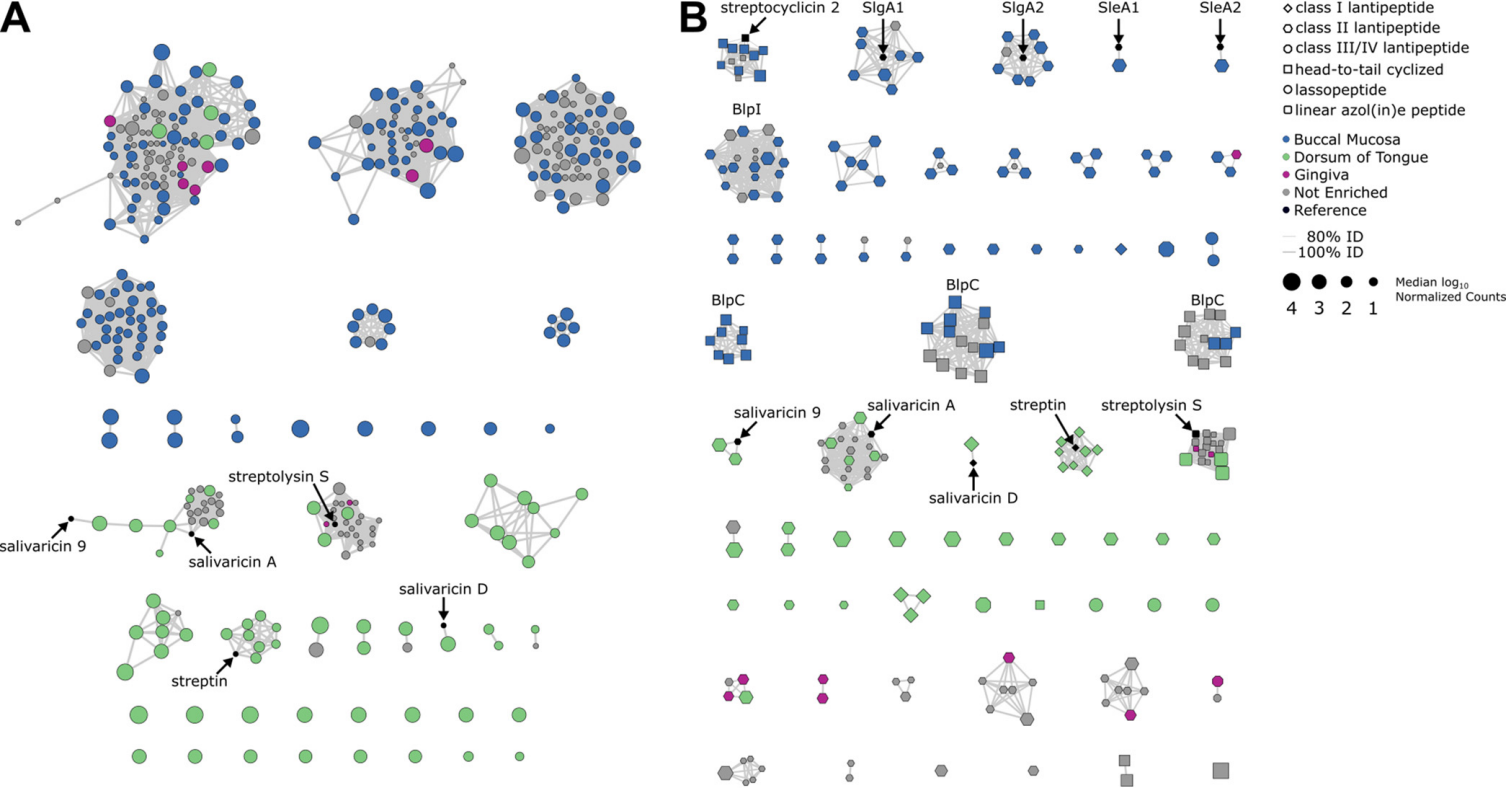
Streptococci from the buccal mucosa and tongue dorsum possess different sets of RiPPs. (A) Subset of the network from Figure 6 showing only RiPP-encoding BGCs predicted from Streptococcus genomes. Black circles represent characterized BGCs from the MIBiG database 1.4 and are labeled with their corresponding RiPP product. The node color and scaling in panel A is consistent with the key in panel B. See Figure 6 legend for specific network details. (B) In this network, each node represents a predicted RiPP core peptide and edges indicate amino acid sequence identity between two nodes (≥80%). The nodes are colored based on ADT site where that BGC was enriched, their shape corresponds to predicted RiPP class, and their size is scaled based on the median log_10_ normalized count value of the BGC in that site (see top right for key). For BGCs that were not enriched in any site, the corresponding node sizes are scaled relative to the median value across all samples and are colored gray. Black nodes representing known RiPP core peptide sequences are labeled and indicated with an arrow (49, 69). The BlpC and BlpI sequence families were identified by comparison to the NR protein sequence database and are labeled.

RiPP-encoding BGCs were the most common BGC class predicted from the genomes of ADT bacteria (Fig. 1A), and were the most enriched BGC class across all ADT sites (Fig. 5). There were 185 BGCs that were enriched in the buccal mucosa, and 183 of these BGCs encoded the biosynthesis of RiPPs or were hybrid clusters that contained two RiPP BGCs (Fig. 6; Fig. S5). In the tongue dorsum, 373 BGCs of all classes were enriched and 142 were RiPP-encoding BGCs. When we investigated the enriched RiPP BGCs from the buccal mucosa, we found that 98% were identified from *Streptococcus* genomes. In contrast, only 49% of the enriched RiPP BGCs in the tongue dorsum were identified from *Streptococcus* genomes, with *Neisseria* and *Veillonella* genomes representing the next largest sources of enriched RiPP BGCs in this site (Fig. 7A; Fig. S5). With few exceptions, the *Streptococcus* RiPP BGCs and BGC families were specifically enriched in either the buccal mucosa or the tongue dorsum (Fig. 7A), which suggested that disparate populations of streptococci, with distinct sets of RiPP-encoding BGCs, colonize these two ADT sites.

Streptococci are common inhabitants of the oral cavity (Fig. 2B), including the buccal mucosa (median: 51% relative abundance [RA] [IQR: 40 to 64% RA]) and tongue dorsum (median: 12% RA [IQR: 8 to 19% RA]). Using an analysis of composition of microbiomes (ANCOM) (61), we determined that Streptococcus gordonii, Streptococcus mitis, Streptococcus oralis, Streptococcus pneumoniae, Streptococcus pseudopneumoniae, Streptococcus sanguinis, and Streptococcus vestibularis were significantly enriched within the buccal mucosa. In contrast, Streptococcus infantis, Streptococcus parasanguinis, and Streptococcus salivarius were significantly enriched in the tongue dorsum (Fig. S7). These distributions are largely consistent with a previous survey of the distribution of streptococci in the oral microbiome, as determined using 16S rRNA gene V3 to V5 region oligotyping, though some of these species (e.g., S. mitis/S. oralis and *S. salivarius*/*S. vestibularis*) are not distinguishable using the V3 to V5 region (62). With the exception of *S. salivarius* and *S. vestibularis*, these streptococci all belong to the mitis group (63), which are known to adhere to nonkeratinized oral mucosa, such as the buccal mucosa (64, 65), and have been reported to colonize buccal cells intracellularly (66). In contrast, *S. salivarius* and *S. vestibularis* are members of the salivarius group and are most commonly isolated from saliva and the tongue dorsum or vestibular mucosa, respectively (63).

As distinct sets of RiPP-encoding BGCs from *Streptococcus* spp. were enriched in the buccal mucosa versus the tongue dorsum (Fig. 7A), we were interested in identifying the specific RiPPs associated with each site. Four RiPP-encoding BGCs or BGC families enriched in the tongue dorsum were similar to characterized BGCs in the MIBiG database (Fig. 7A). Specifically, these BGCs encode the biosynthesis of the RiPPs salivaricin 9 (MIBiG BGC0000547), salivaricin A (MIBiG BGC0000548), and salivaricin D (MIBiG BGC0000549) from *S. salivarius* and streptin (MIBiG BGC0000556) and streptolysin S (MIBiG BGC0000566) from Streptococcus pyogenes (Fig. 7A). In the latter two cases, reads from S. pyogenes were not detected in ADT metagenomes and this bacterium is normally considered to be an opportunistic pathogen and not a regular colonizer of the oral cavity (67). This finding suggests that the streptin and streptolysin S BGCs may be produced by other streptococci that colonize the tongue. To explore this possibility, we analyzed the RiPP-encoding BGCs from 400 *S. salivarius*, 297 S. mitis, 125 S. oralis, 78 S. gordonii, 36 *S. parasanguinis*, 24 S. sanguinis, and 11 *S. infantis* genomes available in GenBank. The BGC for streptin was found in two *S. salivarius* genomes, while the streptolysin S BGC was not detected in any of these 971 Streptococcus species genomes (Fig. S8). The latter result suggests that tongue dorsum metagenomic reads from other streptococci may have spuriously aligned to the streptin and streptolysin S BGCs, instead of their appropriate RiPP BGCs, which may have been absent in the eHOMD genomes.

RiPPs are synthesized as precursor peptides by the ribosome and minimally contain an N-terminal leader peptide and a C-terminal core peptide. The core peptide is then posttranslationally modified and the leader peptide is cleaved to yield the mature peptide, which may or may not be cyclized (68). Therefore, in a complementary approach to determine the RiPPs associated with each BGC, we identified putative open reading frames (ORFs) encoding RiPP precursor peptides in each BGC, predicted the core peptide sequences, and performed sequence alignments against known core peptides for 62 streptococcal-derived RiPPs (49, 69). In addition to the same RiPPs from the tongue dorsum as those described above, we identified the core peptides for streptocyclicin 2 (also called pneumocyclicin) (70), streptolancidin E (also called pneumococcin SP23-BS7, abbreviated SleA1/2) (71), streptolancidin G (also called pneumococcin A, abbreviated SlgA1/2) (72), and bacteriocin-like peptide I (BlpI) as enriched in the buccal mucosa (Fig. 7B). In the case of the streptolancidin E and streptolancidin G, we identified both core peptides, denoted as A1 and A2, encoded within their corresponding BGCs. Furthermore, we also identified multiple Streptococcus BlpC peptide pheromones, which are used by Streptococcus spp. for quorum sensing (73). Notably, 102 of these predicted core peptides, representing 49 families, were not similar to any characterized streptococcal RiPP.

From metagenomic analyses alone, it is not possible to determine how RiPPs contribute to ecological interactions that occur within the oral cavity, but several of the RiPPs that we identified have been characterized previously. The biological activities of RiPPs are varied, but these metabolites often possess antibiotic activity (74) and are sometimes called class I bacteriocins. Indeed, oral streptococci are well known to inhibit the growth of closely related species through the production of RiPPs (75). As examples, salivaricin 9, salivaricin A, salivaricin D, and streptin are each known to broadly inhibit the growth of many, but not all, streptococci and other *Firmicutes* (76–80). Further, while the activity of streptolancidin G from the native producer is unknown, when the core peptides A1 and A2 were fused to the nisin RiPP leader sequence and heterologously expressed in Lactococcus lactis, the resulting RiPPs inhibited Micrococcus flavus (phylum: *Actinobacteria*) (72). However, heterologous expression of RiPPs can cause the resulting peptides to possess aberrant or missing posttranslational modifications (81), which can affect their biological activity. Finally, in addition to mediating bacterial interactions, RiPPs produced by oral streptococci may facilitate interactions with the host. For instance, streptolysin S is a known cytolytic toxin and virulence factor produced by S. pyogenes that is responsible for beta-hemolysis (82, 83). Currently, there are no reported biological activities for streptocyclin 2, streptolancidin E, or any of the other unidentified RiPPs enriched in the buccal mucosa or tongue dorsum. Prior to our analyses, the repertoire of RiPP BGCs contained within S. pneumoniae genomes had been reported (69), but the RiPP-encoding capacity of many other streptococci has thus far been underexplored. The recent discovery of the streptide and streptosactin RiPPs from Streptococcus thermophilus underscores the potential of streptococci to produce bioactive natural products (84, 85).

It currently remains to be determined if the specific sets of RiPPs associated with the buccal mucosa or the tongue dorsum are responsible for mediating interactions with other bacteria that also colonize these distinct sites (Fig. 2B; Fig. 3A). Alternatively, perhaps the oral streptococci produce these RiPPs specifically to outcompete other streptococci, which leads to specific patterns of Streptococcus species colonization across the ADT (Fig. S7). Therefore, future work is necessary to establish connections between these RiPP BGCs, their products, and patterns of biogeography observed in the oral cavity.

### Underexplored genera in the ADT are potential sources of bioactive specialized metabolites.

Among bacteria that colonize the human ADT, previous investigations into specialized metabolism focused largely on the genera *Corynebacterium* (phylum *Actinobacteria*), *Staphylococcus* (phylum *Firmicutes*, class *Bacilli*), and *Streptococcus* (phylum *Firmicutes*, class *Bacilli*). Our preceding analyses and investigation by others (13, 39) demonstrate that other ADT bacteria possess the potential to produce specialized metabolites (Fig. 1 and 6), but many of these organisms have generally been overlooked. Here, we highlight several bacterial genera that may represent promising sources for future natural product discovery.

***Actinomyces*.** *Actinomyces* spp. (phylum *Actinobacteria*) are present in the gingiva (median: 13% RA [IQR: 6.5 to 19% RA]), tongue dorsum (median: 11% RA [IQR: 5.4% to 18% RA]), and buccal mucosa (median: 1.5% RA [IQR: 0.57 to 3.8% RA]). Previous research on *Actinomyces* spp. has focused primarily on their role as opportunistic pathogens and causative agents of the rare disease actinomycosis (86). However, *Actinomyces* are related to *Streptomyces* (phylum *Actinobacteria*), which are well-known and prolific producers of antibiotics and other specialized metabolites (87). Despite this relationship to *Streptomyces*, there is currently only one BGC from *Actinomyces* in the MIBiG database. This BGC is responsible for the production of the antitumor cetoniacytone A, which is produced by an intestinal isolate of *Actinomyces* from the rose chafer beetle (88). In addition, a recent report identified a family of RiPPs called actifensins from *Actinomyces* with broad-spectrum activity against Gram-positive bacteria (89). Beyond these two examples, the Natural Products Atlas (NPAtlas) (90) currently contains 75 compounds reportedly isolated from *Actinomyces*. This compound count is an overestimation, as the name *Actinomyces* was used historically as a misnomer for members of the class *Actinomycetes* (87). Indeed, many of the compounds stated to be sourced from *Actinomyces* in the NPAtlas were derived from *Streptomyces*.

We identified a total of 55 BGCs and BGC fragments from 20 *Actinomyces* genomes (median: 3 BGCs per genome, IQR: 1 to 3.25 BGCs per genome) (Fig. 1B and C). Of these BGCs, 51 and 1 were enriched in the gingiva and tongue dorsum, respectively (Fig. 6; Fig. S5). In particular, 31 RiPP-encoding BGCs from *Actinomyces* were enriched in the gingiva. We detected 20 unique core peptide sequences from these BGCs and searched for related RiPPs, which led us to identify two core peptides with similarity to known RiPPs. The first peptide was from *Actinomyces* sp. HMT 170 strain F0386 and was similar to butyrivibriocin OR79 from Butyrivibrio fibrisolvens (amino acid identity: 84%) (91). The second peptide was from *Actinomyces* sp. HMT 448 strain F0400 and was similar to salivaricin G32 from *S. salivarius* (amino acid identity: 75%) (92). These known RiPPs were previously reported to be active against several *Firmicutes*, including Butyrivibrio fibrisolvens, Lachnospira multiparus, L. lactis, Streptococcus bovis, and S. pyogenes (91, 92). None of the other core peptides from *Actinomyces* were similar to any known RiPP, consistent with this genus being underexplored for specialized metabolite discovery. In addition to RiPPs, the *Actinomyces* genomes also contain 10 BGCs for nonribosomal peptide and 8 BGCs for polyketide metabolites (Table S1).

**FIG 8:**
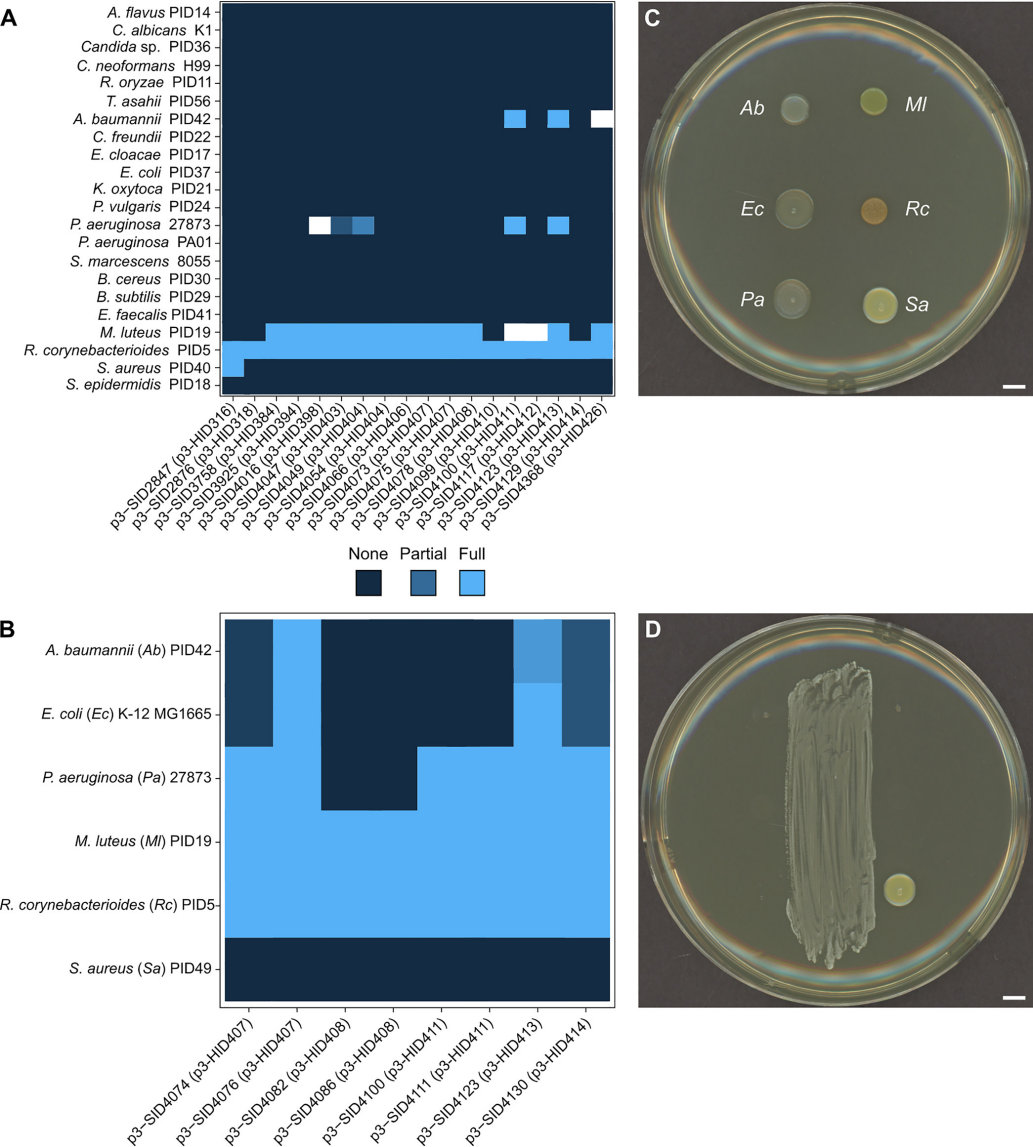
Antibacterial bioactivity of *Actinomyces*. (A) Eighteen *Actinomyces* isolates (horizontal) were monocultured aerobically in BHI agar wells for 1 week before 22 targets (vertical) were spotted adjacent to the *Actinomyces* colony. The colonies were cultured together for 3 days before inhibition of the targets was scored. The heat map displays the inhibition scores of each target when paired with the corresponding *Actinomyces* isolate, as indicated in the key below. Each interaction was repeated with ≥2 replicates. The white cells indicate interactions where replicates were in disagreement or the *Actinomyces* colony overgrew the well before target inoculation. (B) Eight *Actinomyces* isolates (horizontal) were monocultured anaerobically on BHI agar plates for 1 week before six strains of target bacteria were spotted adjacent to the *Actinomyces* colonies. The bacteria were cultured together for 1 day before inhibition was scored. The heat map displays the inhibition scores of each target, as in panel A. Each interaction was triplicated and the average inhibition score is reported. For panels A and B, the volunteer ID is indicated in parentheses and the inhibition scoring system is depicted below panel A. (C) Representative photograph of target bacteria cultured without *Actinomyces*. Each target is indicated by a two-letter abbreviation (see panel B). (D) Representative photograph of *Actinomyces* sp. isolate p3-SID4123 (center of plate) cultured with target bacteria spotted in the same orientation as that of panel C. The scale bars indicate 5 mm.

As a direct test of the inhibitory potential of *Actinomyces*, we first performed targeted isolation from buccal mucosal and tongue dorsum swabs collected from healthy volunteers and from oral swabs and saliva samples that were previously collected as part of the Winning the War on Antibiotic Resistance in Wisconsin (WARRIOR) study (93). We cultured specimens on enrichment medium that we designed and identified *Actinomyces* isolates based on colony morphology and 16S rRNA gene sequence. In total, we tested 18 *Actinomyces* isolates from 16 different donors against a panel of nine Gram-negative bacteria (Acinetobacter baumannii, Citrobacter freundii, Enterobacter cloacae, E. coli, Klebsiella oxytoca, Proteus vulgaris, Pseudomonas aeruginosa [two isolates], and Serratia marcescens), 7 Gram-positive bacteria (Bacillus cereus, Bacillus subtilis, Enterococcus faecalis, Micrococcus luteus, Rhodococcus corynebacterioides, Staphylococcus aureus, and Staphylococcus epidermidis), and 6 fungi (Aspergillus flavus, Candida albicans, *Candida* sp., Cryptococcus neoformans, Rhizopus oryzae, and Trichosporon asahii) (Fig. 8A). All *Actinomyces* isolates tested strongly inhibited the growth of R. corynebacterioides (phylum *Actinobacteria*), which is an opportunistic human pathogen (94), and two-thirds of the isolates strongly inhibited M. luteus (phylum *Actinobacteria*), a generalist that can colonize the ADT and skin (95) (Fig. 8A). For comparison, 43% and 63% of *Streptomyces* isolated from soil inhibited *R. corynebacterioides* and M. luteus, respectively, under similar assay conditions (17). We observed no antifungal activity from any of the *Actinomyces* isolates (Fig. 8A). In a subsequent experiment, we selected a subset of these and other *Actinomyces* isolates and tested their bioactivity when cultured under anaerobic conditions, which are more representative of the *in situ* environment for these bacteria. In this experiment, we included three pairs of *Actinomyces* that were isolated from the same volunteer for biological replication (Fig. 8B). After 1 week in anaerobic chambers, we assessed the ability of *Actinomyces* to inhibit the growth of three Gram-negative bacteria and three Gram-positive bacteria (Fig. 8C) when cocultured aerobically. Consistent with the previous results, we observed full inhibition of M. luteus and *R. corynebacterioides* by all *Actinomyces* isolates (Fig. 8B). Further, we found that 6/8 (75%) of the *Actinomyces* isolates fully inhibited the growth of P. aeruginosa (phylum *Proteobacteria*, class *Gammaproteobacteria*) and two isolates (25%), designated p3-SID4076 and p3-SID4123, inhibited all three Gram-negative bacteria (Fig. 8D), including the ESKAPE pathogens A. baumannii (phylum *Proteobacteria*, class *Gammaproteobacteria*) and P. aeruginosa. We found that p3-SID4074 and p3-SID4076, which were isolated from the same volunteer, reproducibly possessed different biological activities against A. baumannii and E. coli, suggesting that these two isolates are different strains. Together, these results suggest that *Actinomyces* may produce specialized metabolites with activity against both environmental bacteria and human pathogens of interest. Further work is required to characterize the metabolite(s) produced by *Actinomyces* and identify their mechanisms of action.

***Aggregatibacter*.** Members of the genus *Aggregatibacter* (phylum *Proteobacteria*, class *Gammaproteobacteria*) are low abundant opportunistic pathogens associated with the gingiva (median: 0.7% RA [IQR: 0.18 to 1.8% RA]) that can cause aggressive periodontitis (96) and infective endocarditis (97). We identified a single family of 13 predicted thiopeptide RiPP-encoding BGCs from *Aggregatibacter* spp. that was enriched in the gingiva (Fig. 6; Fig. S5). These BGCs contained the essential YcaO-like cyclase and radical SAM enzymes, but using both automated and manual searches, we were unable to identify any core peptide sequences. Bacteriocin activity has previously been reported from Aggregatibacter actinomycetemcomitans (98, 99), but it is unlikely to be associated with this thiopeptide family, as the activity was labile and susceptible to proteolysis. In addition, we identified 36 aryl polyene BGCs from A. actinomycetemcomitans genomes, but these BGCs were not enriched at any ADT site.

***Cutibacterium*.** Cutibacterium acnes (formerly Propionibacterium acnes, phylum *Actinobacteria*) is abundant in the external naris (median: 38% RA [IQR: 18 to 62% RA]) but is not typically found at other ADT sites. This bacterium is associated with acne vulgaris, but recent evidence suggests that development of this condition is complex and involves multiple factors (100). We identified four BGC families from this organism that were enriched in the external naris (Fig. 6; Fig. S5). There were 12 bacteriocin BGCs in two bacteriocin families, 11 BGCs in one NRPS family, and 3 BGCs in the recently described thiopeptide cutimycin family (101).

***Neisseria*.** Prior work on *Neisseria* spp. (phylum *Proteobacteria*, class *Betaproteobacteria*) has focused primarily on the pathogens Neisseria gonorrhoeae and Neisseria meningitidis as the cause of gonorrhea and bacterial meningitis, respectively. However, *Neisseria* is a diverse genus, and several species are common members of oral microbiomes, including the tongue (median: 10% RA [IQR: 1.6 to 23% RA]), gingiva (median: 6.2% RA [IQR: 2.6 to 13% RA]), and buccal mucosa (median: 2.5% RA [IQR: 0.86 to 6.0% RA]) (102). Although there are currently no reported BGCs in the MIBiG database or compounds in the NPAtlas from *Neisseria*, we identified 166 BGCs and BGC fragments from 35 *Neisseria* genomes (median: 4 BGCs per genome [IQR: 4 to 5.5 BGCs per genome]) (Fig. 1B and C). In the tongue dorsum and gingiva, 25 and 15 terpene-encoding BGCs from 13 *Neisseria* species were enriched, respectively (Fig. 6; Fig. S5). Both N. gonorrhoeae and N. meningitidis genomes are reported to contain *hpnD*, which encodes a head-to-head prenyl synthase that condenses two molecules of farnesyl diphosphate to form presqualene diphosphate (103). In other microbes, presqualene diphosphate is converted into terpenoid products, such as the pigment staphyloxanthin in S. aureus or the sterol-like hopanoids (104), but the presence of squalene-derived metabolites has not been reported from *Neisseria* and efforts to reconstitute the other synthases *in vitro* have not succeeded (103).

In addition to aryl polyenes and terpenes, bacteriocins are the other major class of BGCs possessed by *Neisseria* and enriched in oral cavity microbiomes. We identified 32 bacteriocin-encoding BGCs from 13 *Neisseria* species. These BGCs formed two families that were enriched in the tongue dorsum and gingiva (Fig. 6; Fig. S5). Bacteriocin production has previously been reported from N. gonorrhoeae (105, 106) and N. meningitidis (107–110), but the molecules responsible for this activity have never been identified and the genetic loci have not been confirmed. Therefore, these uncharacterized BGCs may be responsible for the previously reported bacteriocin activity from N. gonorrhoeae and N. meningitidis. Further, a recent report has demonstrated that Neisseria mucosa strains inhibit N. gonorrhoeae in coculture. Organic solvents have been used to extract antigonoccal activity from N. mucosa cultures, but the identities of these putative antibiotics are not currently known (111).

### Conclusions.

The ADT is the main threshold that microbes pass to enter the human body. Bacteria that colonize this system must compete with invaders and other members of the microbiota. We find that human ADT microbiomes possess extensive potential for the biosynthesis of specialized metabolites and other bioactive natural products and demonstrate bioactivity from *Actinomyces*, a bacterial genus that has been underexplored for specialized metabolism. The vast majority of the BGCs from these bacteria remain uncharacterized, but given examples of specialized metabolites mediating competitive interactions in the nasal cavity through siderophore (31) and antibiotic (32) activities, it is likely that these BGCs encode metabolites that affect interactions between members of the ADT microbiota. These interactions may contribute to population dynamics and spatial patterning of the microbiota across the ADT. Indeed, we demonstrate that ADT sites cluster together based on both BGC and bacterial community composition and are each enriched with a distinct set of BGCs, despite often being colonized by closely related bacteria. Together, these results provide evidence for biogeography of specialized metabolism within the human ADT. Further, while our analyses identified broad-scale biogeographical signatures, submicron biogeography in the ADT is critical for understanding how BGCs shape interactions and biogeography within the ADT (1, 112). A limitation of our present study is that detection by metagenomic sequencing does not indicate that the corresponding BGC is expressed. The nasal cavity is the only ADT site with available metatranscriptomic data from the iHMP (41) and has been used previously to detect expression of the dehydroxynocardamine BGC (31). However, expression of a given BGC also does not confirm that the cognate specialized metabolites are produced *in situ*. Therefore, future studies that combine complementary approaches, including genomics, metagenomics, transcriptomics, bacteria interaction assays, and novel analytical chemistry techniques, such as *in situ* mass spectrometry (113), will enable the discovery of new specialized metabolites, inform our understanding of ecology and biogeography of the ADT, and provide insight into the role these metabolites play *in situ* (31, 32, 39, 85, 101, 114, 115).

## MATERIALS AND METHODS

### Identification of BGCs from ADT bacteria.

To build a database of BGCs from bacteria that colonize the human ADT and environmental bacteria that are found transiently in the ADT, we first downloaded 1,527 bacterial genome sequences, representing 162 unique genera and 451 unique species, from the eHOMD V9.03 (30). We classified bacteria as being from the ADT (eHOMD habitat was listed as “Nasal,” “Nasal,Oral,” or “Oral”) or from the environment (eHOMD habitat was listed as “NonOralRef,” “Skin,” “Unassigned,” or “Vaginal”). We identified 3,895 BGCs (Table S1) using antiSMASH 4.2.0 (36) with the following parameters: --clusterblast, --knownclusterblast, --smcogs.

### Sampling ADT metagenomes.

We downloaded the raw reads for 1,424 whole metagenome shotgun sequencing samples from the buccal mucosa, dorsum of tongue, external naris, gingiva, hard palate, nasal cavity, palatine tonsil, throat, and saliva from the iHMP (41). In total, 235 individuals were sampled between 1 and 16 times (median: 6 samples per individual [IQR: 4 to 8 samples per individual]) as part of the “Healthy Human Subjects” and “prediabetes” iHMP studies (Table S2). For any given visit, individuals were sampled at 1 to 7 sites (median: 3 sites [IQR: 2 to 4 sites]). For all analyses, we treated each individual metagenome as an independent sample.

### Alignment of metagenomic reads to BGCs.

We used hmmscan from HMMER 3.1b2 (http://hmmer.org/) to identify ORFs that are commonly associated with BGCs but encode proteins that are not involved in the biosynthesis of specialized metabolites (13). During the hmmscan, we discarded four BGCs that were erroneously called. Using kallisto 0.46.0 (116), we built a length 31 *k*-mer index that contained all ORFs from the BGCs predicted by antiSMASH, except for the nonbiosynthetic ORFs. Among these ORFs, there were 40,045 non-ACGT characters that were replaced by pseudorandom nucleotides. In total, the target de Bruijn graph contained 488,883 targets and 31,840,424 *k*-mers. We used fastp 0.20.0 (117) for quality control and preprocessing of the raw reads downloaded from the iHMP. We used kallisto quant to pseudoalign the processed reads onto this index. For subsequent analyses, we aggregated all estimated read counts for all ORFs into a single estimated read count per BGC. We removed BGCs that were not detected in any of the metagenome samples (21 BGCs from ADT bacteria and 274 BGCs from environmental bacteria). We rounded the estimated counts to the nearest integer value and added one pseudocount to each BGC in each sample. We normalized the estimated counts for each BGC using the size factor (118). To perform hierarchical clustering, we used Ward’s minimum variance method on the squared Euclidean distances between the metagenome samples in R 4.0.0 (119).

### Metagenome composition.

To determine the bacterial community composition of metagenomes from the iHMP, we used MetaPhlAn 3.0 (120) with the following parameters: --ignore_archaea and --ignore_eukaryotes. MetaPhlAn estimates the RA of microbial taxa in metagenomes by calculating the coverage of metagenomic reads over clade-specific marker genes.

### NMDS and ANOSIM.

We used the vegan package 2.5–6 (121) in R to perform NMDS and ANOSIM. To generate NMDS plots, we used the metaMDS function with the following parameters: k = 2, trymax = 100. To test for an effect of ADT site on species and BGC composition in the metagenome samples, we performed an ANOSIM with the following parameters: distance = “bray,” permutations = 10000. For both NMDS and ANOSIM of species composition, we filtered out species that were present in metagenomes at <0.1% RA or at 100% RA and species present in <25 metagenomes.

### Bacterial community and BGC diversity comparisons.

We used H′ (122) to calculate the alpha diversity of species-level bacterial communities and BGCs in ADT metagenome samples. For bacterial communities, we used the RA determined by MetaPhlAn. For BGCs, we determined proportions by dividing the number of reads that aligned to each cluster by the total number of reads in a given metagenome. We determined significant differences in H′ across ADT sites by a Kruskal-Wallis one-way analysis of variance (ANOVA) followed by a *post hoc* Dunn’s test with Benjamini-Hochberg correction for multiple hypothesis testing (*α* = 0.05) using the fisheries stock analysis (FSA) (123) and Dunn’s test (124) packages in R. We calculated ε^2^ (125) using the rcompanion package (126) in R to measure effect size of ADT site on H′ metrics.

### Differential BGC abundance testing.

To identify BGCs that were specifically enriched in microbiomes from different sites, we used DESeq2 1.26.0 (127) in R to compare a given site against all other sites in the data set. For this analysis, we selected only sites with ≥200 metagenome samples. We considered a BGC to be enriched in a specific site if it was detected at a ≥4-fold difference over all other sites with a Wald’s test adjusted *P* value of <1.0e−08.

### BGC similarity network.

To identify relationships among the 3,895 valid ADT BGCs, we used BiG-SCAPE 1.0.1 (48) with the following parameters: --cutoffs 0.3, --include_singletons, --mix. In addition, we included BGCs in the MIBiG database 1.4 (49) in our network analysis using the following parameter: --mibig. During generation of the network, four additional low-quality BGCs were excluded. We visualized the network using Cytoscape 3.8.0 (128).

### ANCOM.

We used ANCOM v2.1 (61) in R to detect species that were differentially abundant between ADT microbiomes. ANCOM performs pairwise comparisons of the ratio of each taxon to every other taxon and determines if these ratios differ between different types of microbiomes (e.g., different ADT sites) with Benjamini-Hochberg correction for multiple hypothesis testing. The outcome of an ANCOM analysis is the *W*-statistic for each taxon, which is the number of pairwise comparisons in which the null hypothesis was rejected (*α* = 0.05). Prior to ANCOM, we estimated the number of reads for each taxon by multiplying the relative proportions estimated by MetaPhlAn 3.0 by the total number of reads after quality trimming with fastp. We filtered out taxa that were present in <10 metagenomes or represented by <1,000 estimated reads. Metagenomes that contained <10,000 total reads were also excluded from the analysis. We considered taxa to be differentially abundant between ADT sites if the absolute value of the mean difference of their center log ratio (CLR)-transformed relative abundances between sites was >0.5 and the corresponding *W*-statistic was in the 90th percentile.

### Identification of RiPP core peptides.

To identify putative RiPP core peptides, we first extracted and translated all ORFs ≤450 nucleotides from RiPP-encoding BGCs predicted by antiSMASH. Next, we used NLPPrecusor from DeepRiPP (129) to classify each peptide either as non-RiPP-encoding or into specific RiPP classes (score of ≥0.75). For peptides classified as RiPPs, we removed the predicted leader sequence and used DIAMOND BLASTP (130) to align the core peptides against each other, previously characterized RiPPs from *Streptococcus* genomes (49, 69) (query length of 100%, query ID of ≥75%), and the NR protein sequence database. We visualized the network of related RiPPs using Cytoscape 3.8.0.

### Isolation and culture of *Actinomyces*.

Informed consent was obtained from volunteers, and the Human Subjects Committee at the University of Wisconsin-Madison approved the study (institutional review board [IRB] approval number 2020-1027). Each volunteer self-swabbed their buccal mucosa and tongue dorsum using sterile flocked eSwabs (Copan Diagnostics) for approximately 15 s over a 3 cm^2^ area. After sampling, the volunteers immersed the swabs in 1 ml modified liquid Amies medium (Copan Diagnostics) for transport to the laboratory. Saliva was collected into a sterile tube, and the buccal mucosa and tonsils were swabbed (designated “oral swabs”) from participants of the WARRIOR study (93) during clinic visits and processed in our laboratory (IRB approval number 2018-1578). We serially diluted the samples in phosphate-buffered saline and inoculated 100 μl onto *Actinomyces* enrichment medium 1 plates (AEM1; 3.7% [wt/vol] BBL brain heart infusion [BHI; BD], 0.37% [wt/vol] sodium bicarbonate, and 1.5% [wt/vol] Bacto agar [BD], with the following additions after autoclaving and cooling the medium to 55°C: 0.005% [wt/vol] lithium mupirocin [Sigma-Aldrich], 0.003% [wt/vol] nalidixic acid [Dot Scientific], 0.1% [vol/vol] Tween 80 [Sigma-Aldrich], and 0.001% [vol/vol] vitamin K_1_ [TCI]). We incubated AEM1 plates for 7 days at 37°C in a GasPak jar containing carbon dioxide (CO_2_) generators (BD). We selected ≥2 colonies of each distinct morphotype per sample and passaged the isolates anaerobically on BHI plates at 37°C until we obtained pure cultures. We inoculated BHI broth with the bacterial isolates and incubated these cultures statically at 37°C until there was sufficient bacterial density for subsequent experiments (typically 2 days to 1 week). All bacterial isolates were cryopreserved at −80°C in 25% (vol/vol) glycerol (Dot Scientific).

### Colony PCR and identification of bacterial isolates.

To identify bacterial isolates, we sequenced the 16S rRNA gene. Briefly, we combined 20 μl of resuspended liquid cultures with 20 μl of 0.04 N sodium hydroxide and incubated the samples for 5 min at 95°C. Afterwards, we centrifuged the samples for 5 min at 21,130 × *g* and used 1 μl of the supernatant as the template for PCR amplification of the 16S rRNA gene using the universal 27F (5′-AGAGTTTGATCMTGGCTCAG-3′) and 1492R (5′-CGGTTACCTTGTTACGACTT-3′) primers (131). We verified amplification of PCR products using electrophoresis with Tris-acetate-EDTA (TAE) gels (40 mM Tris base, 20 mM acetic acid, 1 mM EDTA disodium salt, 1% [wt/vol] agarose). The PCR products were cleaned and sequenced with the Sanger method using the 27F primer at Functional Biosciences (Madison, WI). We identified isolates to the genus level using the eHOMD 16S rRNA sequencer identifier and ribosomal database project classifier (30, 132).

### Coculture plate inhibition assays.

To assess the inhibitory activity of *Actinomyces* isolates, we used coculture plate inhibition assays as described previously (17, 31, 133). We diluted static liquid cultures of the bacterial isolates (see above) to an optical density at 600 nm (OD_600_) of 0.5 and spotted 3 μl on one half of a well (diameter, 2.4 cm) containing 3 ml of BHI agar on a 12-well plate (Greiner Bio-One). We aerobically incubated the plates at 37°C for 1 week. We diluted overnight cultures of the target strains to an OD_600_ of 0.5 using fresh lysogeny broth (LB; 1% [wt/vol] tryptone [Difco], 0.5% [wt/vol] yeast extract [Bacto], 0.5% [wt/vol] sodium chloride [Sigma-Aldrich]) or yeast-peptone-dextrose (YPD; 1% [wt/vol] yeast extract [Bacto], 2% [wt/vol] peptone [Bacto], 2% [wt/vol] dextrose [VWR]) for bacteria and yeasts, respectively. We maintained filamentous fungi as spore stocks at −80°C, which we thawed and diluted 10-fold in YPD before use. Subsequently, we spotted 2 μl of each target adjacent to the established bacterial colonies and incubated the plates at 28°C. After 3 days of coculture, we scanned the plates and scored the inhibition phenotype for each interaction pair. Wells with contamination or overgrowth were not scored. All interactions were tested with ≥2 replicates with consistent results. We scored the plates for inhibition after overnight incubation. An inhibition score of 0 indicated no inhibition, a score of 1 indicated partial inhibition, and a score of 2 indicated full inhibition. All interactions were tested with ≥2 replicates, with consistent results.

For subsequent assays to test the bioactivity of *Actinomyces* isolates when cultured anaerobically, we diluted liquid cultures to an OD_600_ of 0.5 and spread 50 μl in an ∼6 cm by ∼2 cm wide line in the center of a 25 ml BHI agar plate (diameter 8.6 cm) using an inoculating loop. We then incubated the plates at 37°C in a GasPak jar with CO_2_ generators, as described above. After 1 week, we removed the plates from the GasPak jar and spotted 5 μl of the target bacteria at an OD_600_ of 0.5 approximately 0.5 cm away from the line of *Actinomyces*. Once the spots dried, we aerobically incubated the plates at 37°C. All interactions were tested in triplicate, scored, as described above, and averaged.

### Statistical analysis and data visualization.

All statistical analyses were performed in R, and the packages are described in the preceding sections. We generated graphs using ggplot2 (134), heat maps using ComplexHeatmap (135), and volcano plots using EnhancedVolcano (136), with some cleanup and figure assembly in InkScape.

### Data availability.

The genome sequences used for BGC prediction were downloaded from the eHOMD (http://www.ehomd.org/), and all raw metagenome reads were downloaded from the iHMP data portal (https://portal.hmpdacc.org/). An R Markdown file and all Python scripts necessary to replicate this work are available here: https://github.com/reedstubbendieck/adt_bgcs. The data sets used for all analyses and figure generation are available here: https://doi.org/10.6084/m9.figshare.14217326.
