# Supplementary material for: Biogeography of Bacterial Communities and Specialized Metabolism in Human Aerodigestive Tract Microbiomes
Source: Microbiol Spectr. 2021 Oct 27;9(2):e01669-21. doi: 10.1128/Spectrum.01669-21 (PMC8549736; doi:10.1128/Spectrum.01669-21)
Supplement: SUPPLEMENTAL FILE 3 — Supplemental material. Download Spectrum.01669-21-s0003.pdf, PDF file, 3.3 MB [file spectrum.01669-21-s0003.pdf]

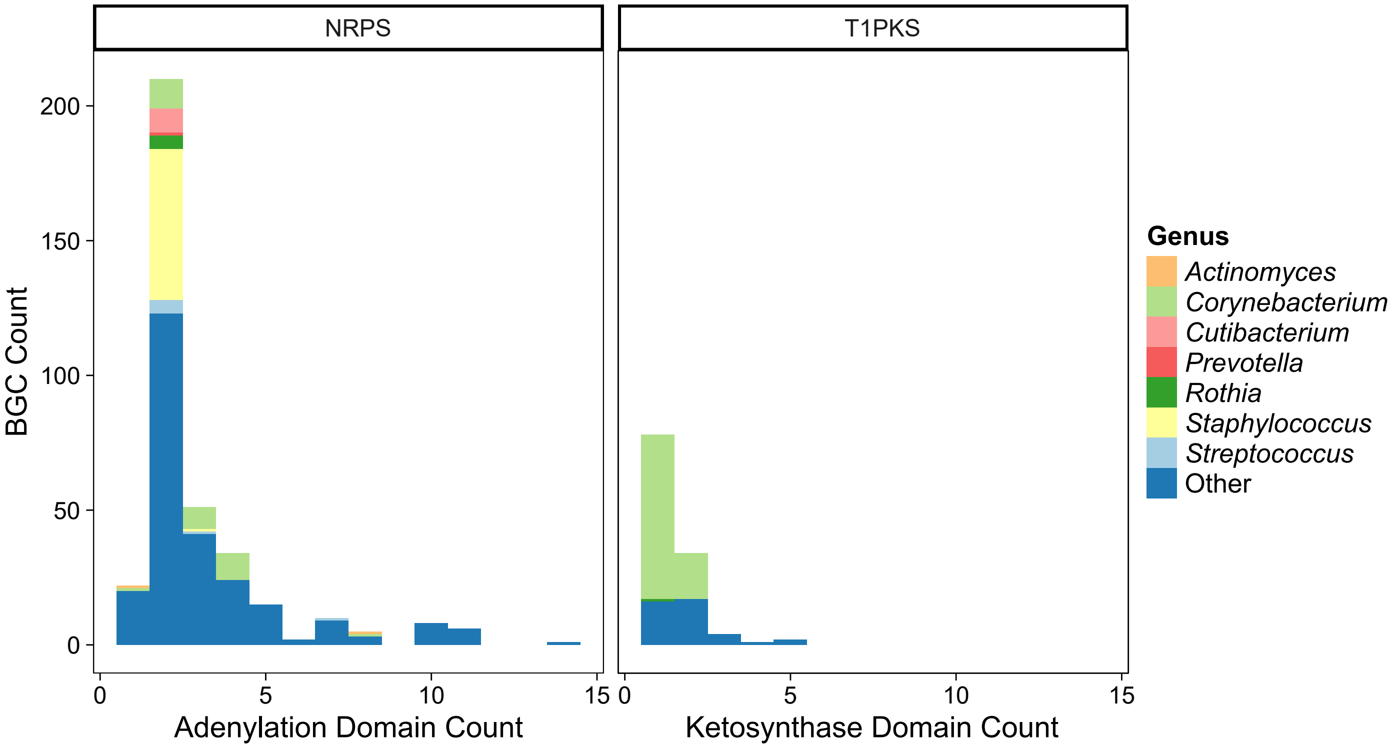

FIG S1 NRPS and type I PKS BGCs from ADT bacteria encode low numbers of biosynthetic modules. The histograms show the number of adenylation (AMP-binding) and ketosynthase (PKS_KS) domains for NRPS and type I PKS BGCs, respectively, predicted from eHOMD genomes. The histogram bars are colored by bacterial genus, as indicated by the key. BGCs that were located on contig edges were excluded from this analysis.

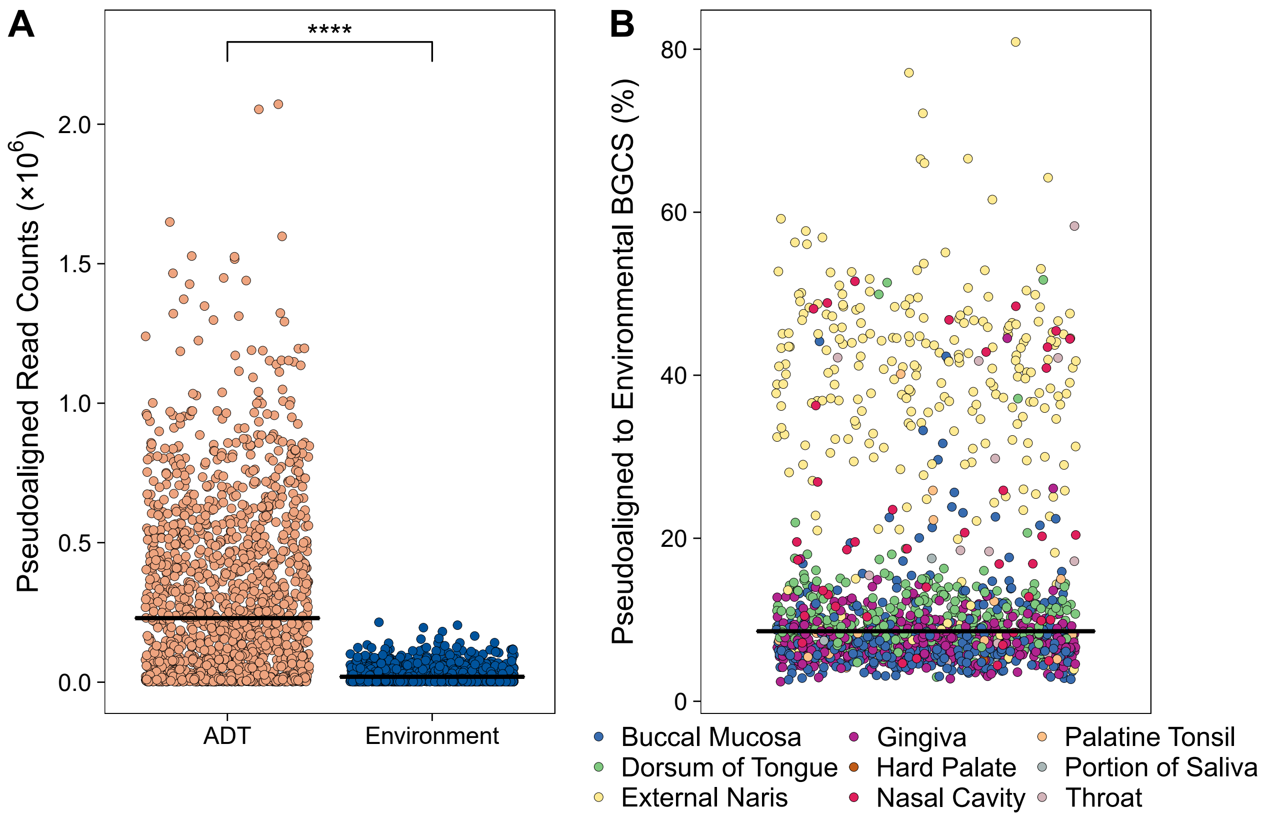

FIG S2 The majority of reads from ADT metagenome samples map to BGCs from ADT bacteria versus BGCs from environmental bacteria. **(A)** The plot indicates the number of reads from 1424 ADT metagenomes that mapped to BGCs identified from ADT (eHOMD habitat listed as "Nasal", "Nasal,Oral", or "Oral") and environmental (eHOMD habitat listed as "NonOralRef", "Skin", "Unassigned", or "Vaginal") bacteria. The horizontal black bars represent the median count of reads pseudoaligned to ADT or environmental BGCs. Points have been jittered to avoid overplotting. ****, indicates a significant difference in the signed differences between counts mapping to ADT BGCs and environmental BGCs. **(B)** The plot indicates the percentage of reads from 1424 ADT metagenome samples that pseudoaligned onto BGCs from environmental bacteria versus BGCs from ADT bacteria. Each point represents a single metagenome sample and is colored based on ADT site, as indicated in the key. The horizontal black bar represents the median percentage of reads that map to environmental BGCs across all metagenome samples. Points have been jittered to avoid overplotting.

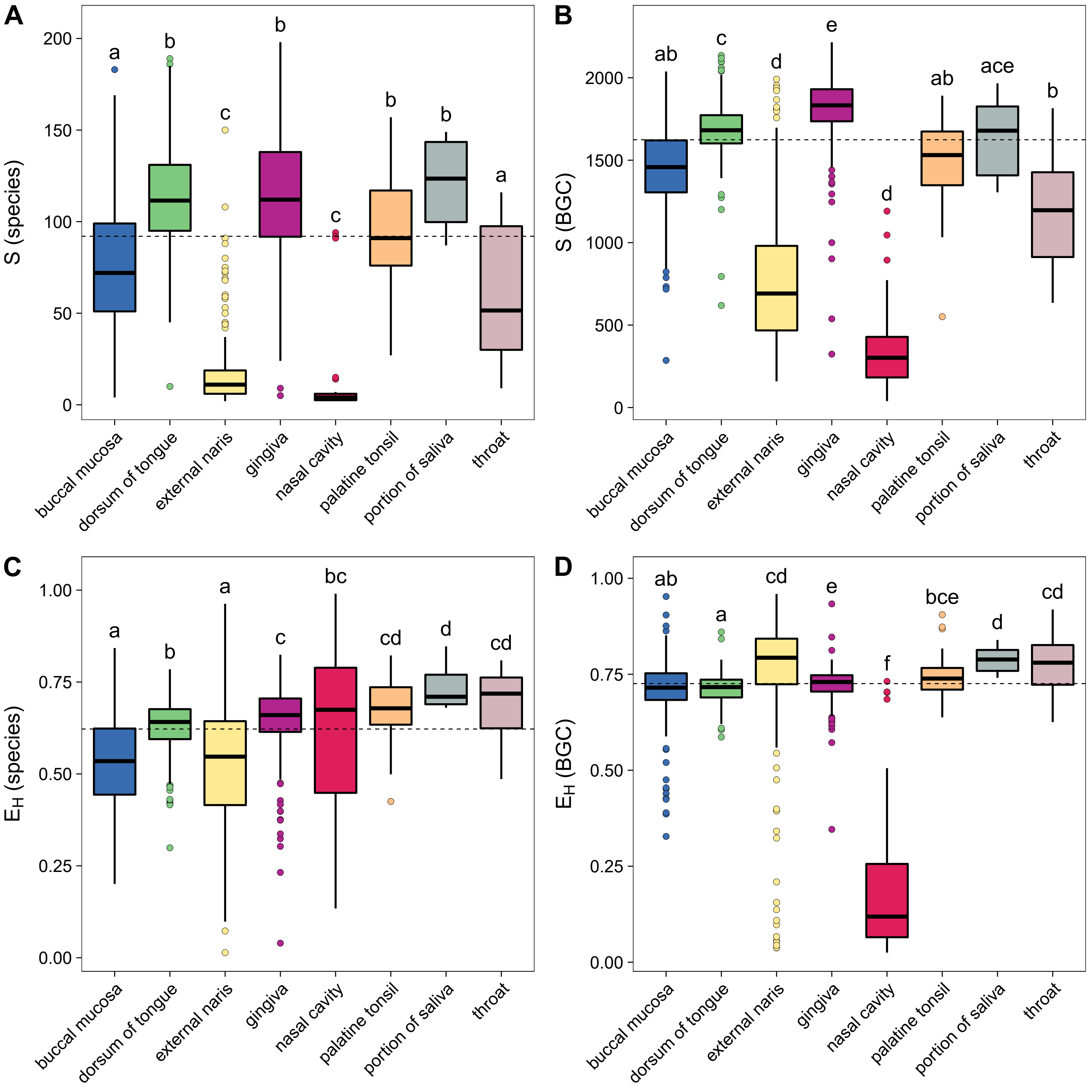

FIG S3 Bacterial species and BGC richness and evenness vary across the ADT. Box plots for richness (S) and Shannon's equitability (E_H) for species-level bacterial community and BGC composition in the ADT. The S value is the total number of distinct **(A)** species or **(B)** BGCs detected in each metagenome sample. The E_H value represents how evenly **(C)** species or **(D)** BGCs are distributed in each metagenome sample, where a value of 1 indicates complete evenness. The upper and lower bounds of the box plots indicate the 75th and 25th percentiles, respectively. The horizontal black bars indicate the medians. The whiskers extend from the bounds of the box to the largest and smallest values that are no further than ±1.5 × the IQR. All outliers that occur outside this range are shown as points. ADT sites that share letters are not significantly different (α=0.05). The horizontal dashed lines indicate the overall median value for each panel. For these analyses samples with $H'_{species}$ = 0 were removed.

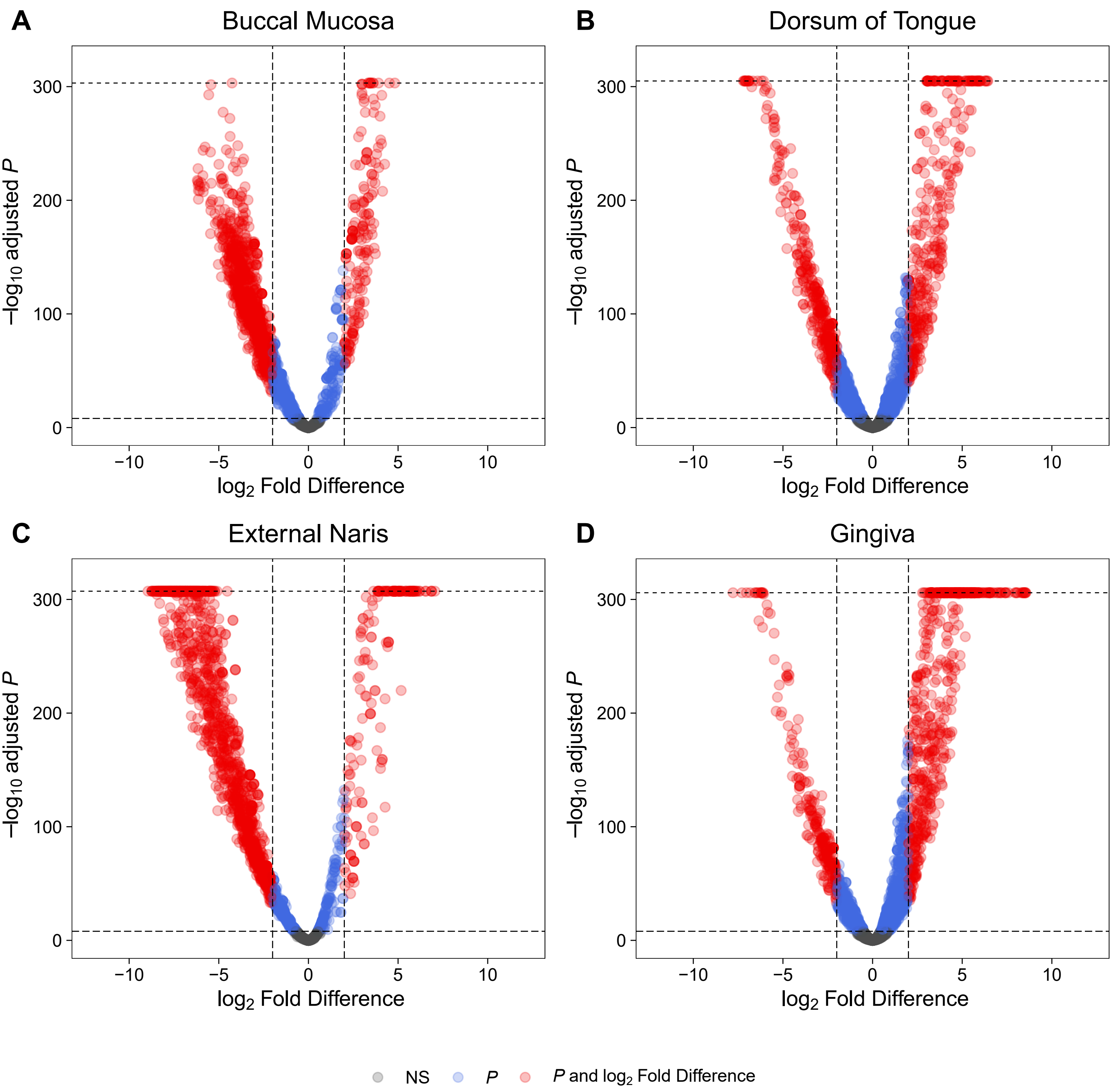

FIG S4 BGCs abundance varies across ADT microbiomes. The volcano plots indicate the enrichment of BGCs in specific sites across the human ADT: **(A)** the buccal mucosa, **(B)** the dorsum of the tongue, **(C)** the external naris, and **(D)** the gingiva. Each point represents a BGC and is colored based on the *P*-value and fold change thresholds as indicated by the key. The horizontal dashed line on the bottom and top of the plots indicate the Wald's test adjusted *P*-value threshold of 10e-08 and the theoretical minimal *P*-value from the corresponding enrichment analysis (0.1 × the lowest non-zero enriched *P*-value), respectively. The vertical dashed lines on the left and right of the plots indicate the fold change cutoffs for BGCs that are decreased and increased, respectively, in the given site over all other sites in the ADT. NS, not significant.

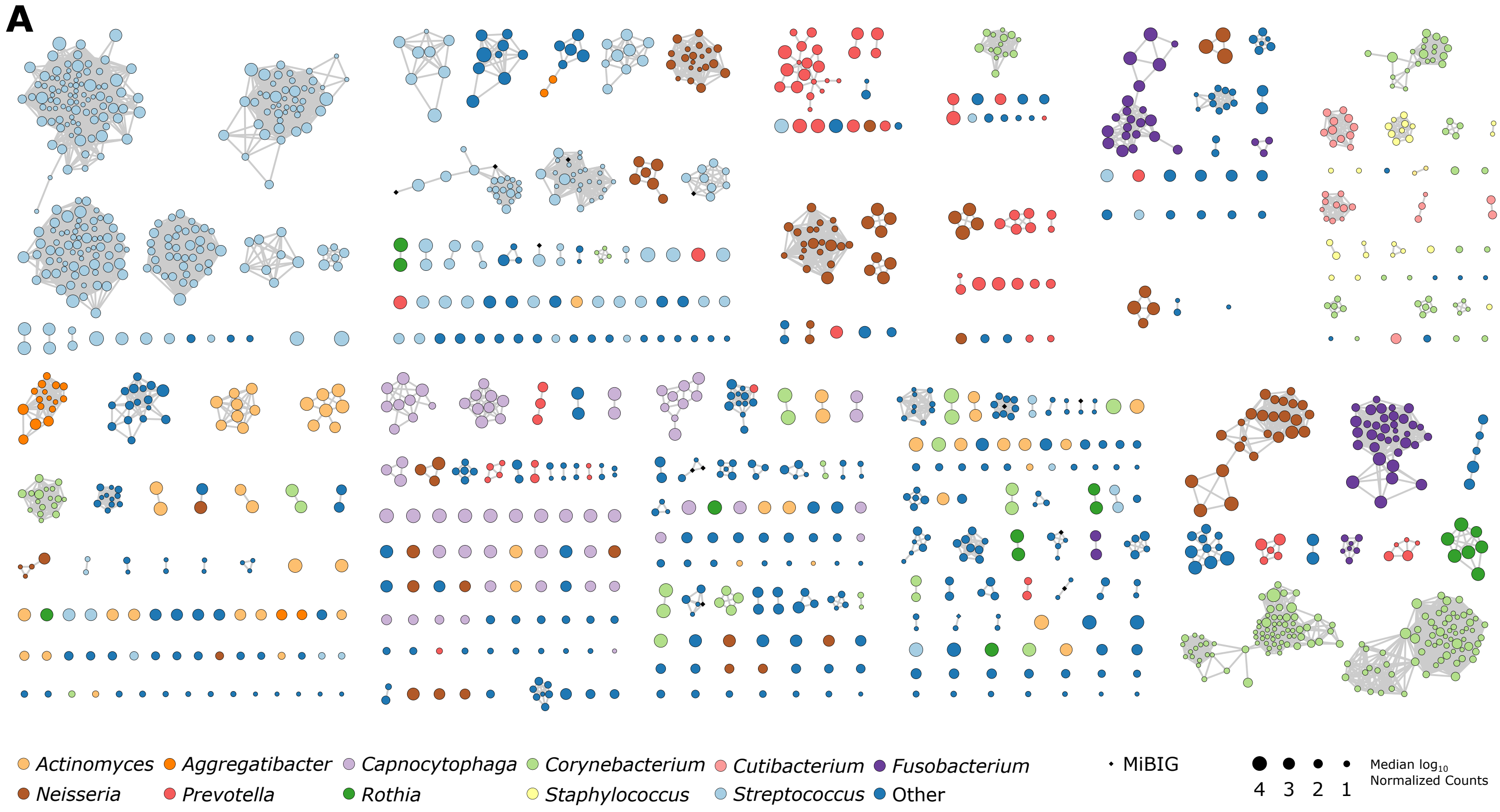

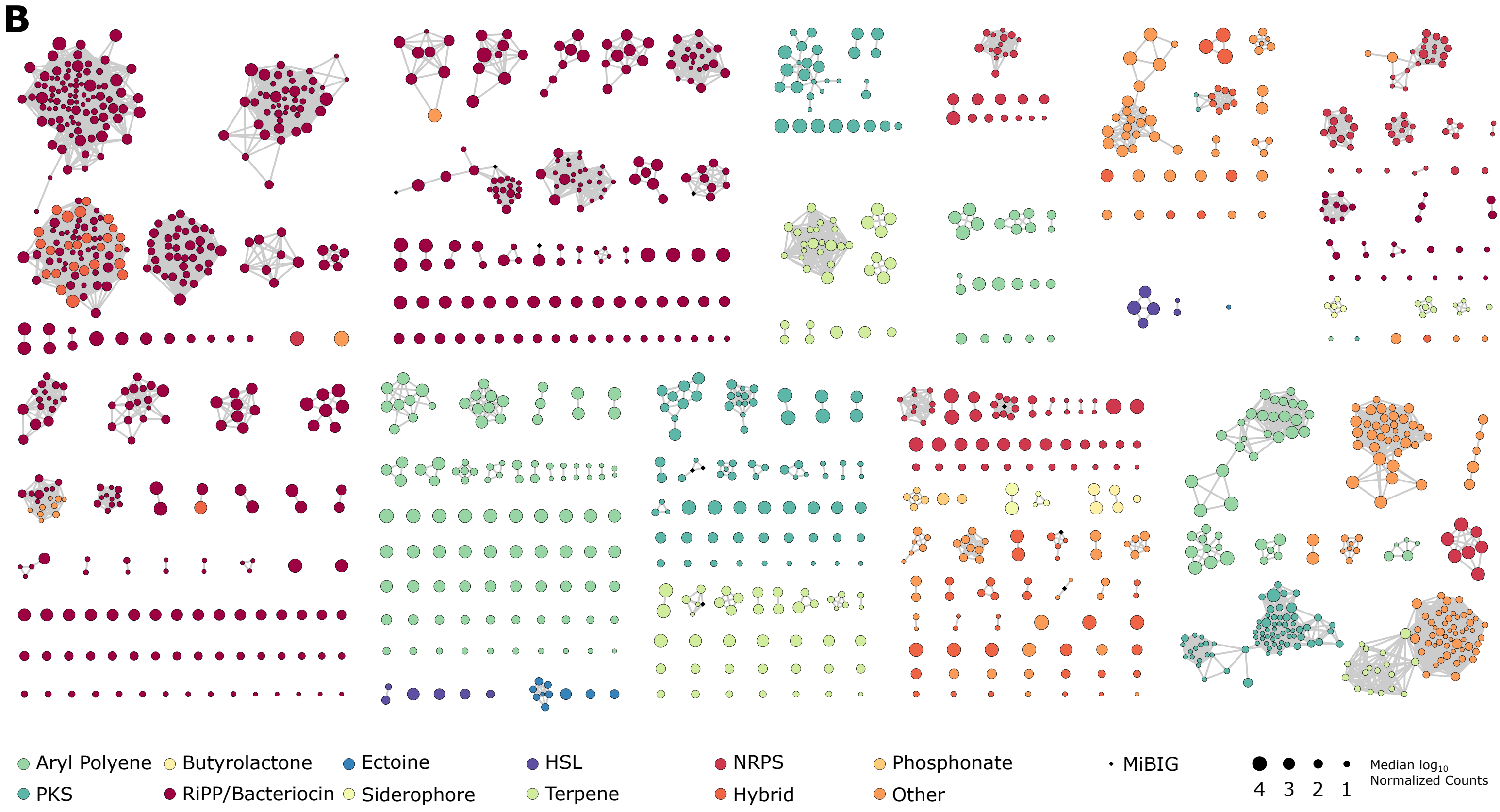

FIG S5 Identification of BGC families associated with specific ADT microbiomes. In this network, each node represents a single BGC and edges indicate weighted pairwise distances between the corresponding BGCs. The nodes are colored based on the **(A)** bacterial genus from which the corresponding BGC was identified and **(B)** BGC type, respectively, and their size is scaled based on the median normalized count value of the BGC in for their enriched ADT site (Fig. 6) (see black circles in the bottom right for key). For BGCs that were not enriched in any site, the corresponding node sizes are scaled relative to the median value across all samples. Black diamonds represent characterized BGCs from the MIBiG database 1.4. For clarity, BGC families with few or no enriched clusters were removed.

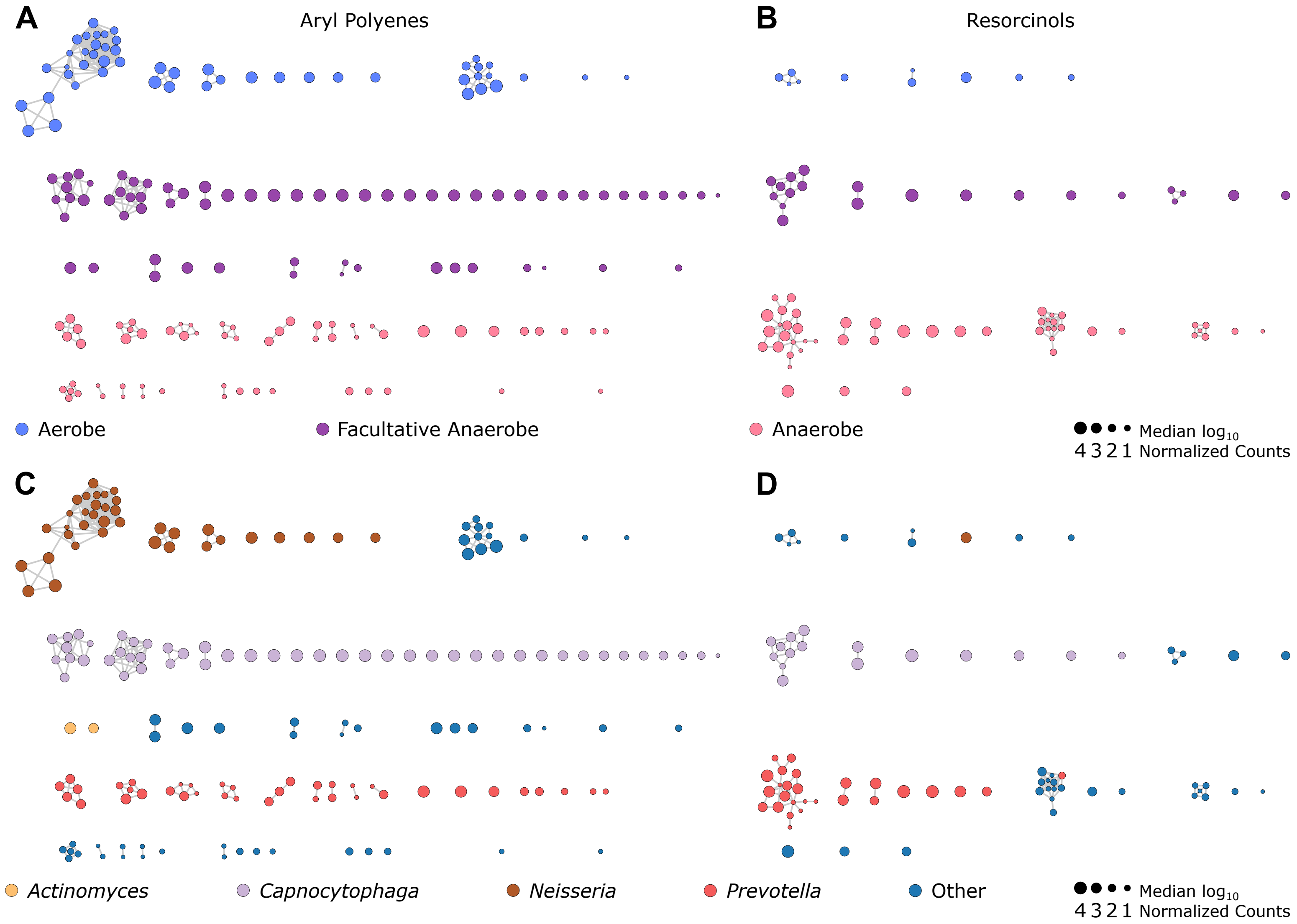

FIG S6 Aryl polyene and resorcinol BGCs enriched in the gingiva and tongue dorsum are associated with anaerobic and facultative anerobic bacteria. In this network, each node represents a single BGC and edges indicate weighted pairwise distances between the corresponding BGCs. The nodes are colored based on the (**A**, **B**) oxygen requirements and (**C**, **D**) genus of bacteria from which the corresponding BGC was identified. The size of the nodes size is scaled based on the median $\log_{10}$ normalized count value of the BGC in its enriched site (see black circles in the bottom right for key).

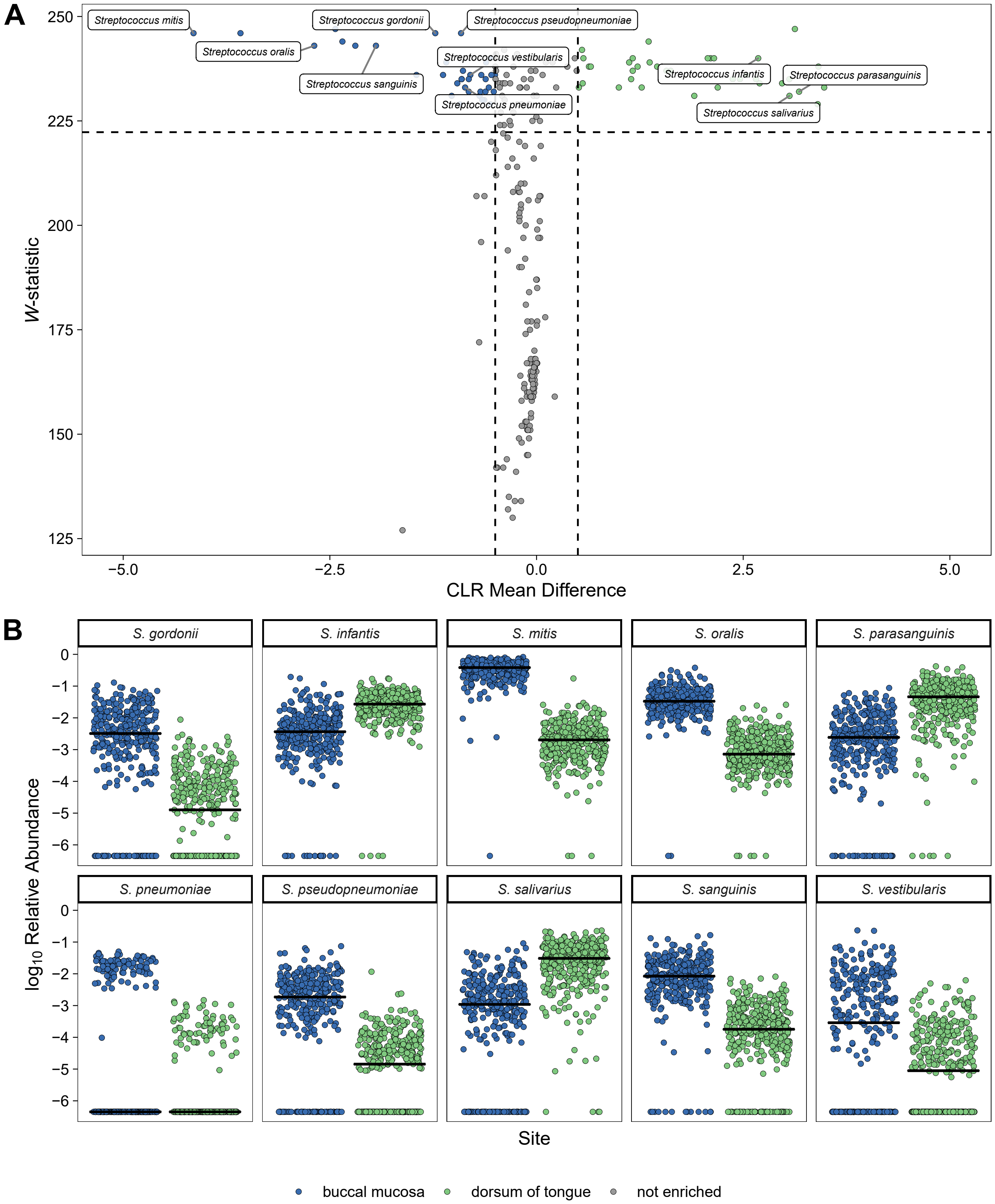

FIG S7 Different species of *Streptococcus* colonize the buccal mucosa and tongue dorsum. (**A**) ANCOM analysis of species that are differentially abundant between buccal mucosa and tongue dorsum metagenomes. Each point represents a single bacterial species and is colored according to the enriched ADT site. The vertical dashed lines indicate ±0.5 of the mean difference of center log ratio-transformed relative abundances between ADT sites. The horizontal line indicates the 90th percentile value for the *W*-statistic (see Materials and Methods). Named species of *Streptococcus* that are differentially abundant between ADT sites are labeled. (**B**) $\log_{10}$ relative abundance of *Streptococcus* spp. across the buccal mucosa and dorsum of tongue. Each point represents a single metagenome sample and is colored according to the ADT site. Before $\log_{10}$ transformation a pseudocount equal to the 0.5 × lowest non-zero relative abundance was added to each relative abundance value. The horizontal black bars represent the median $\log_{10}$ relative abundance of each genus per site. Points have been jittered to avoid overplotting.

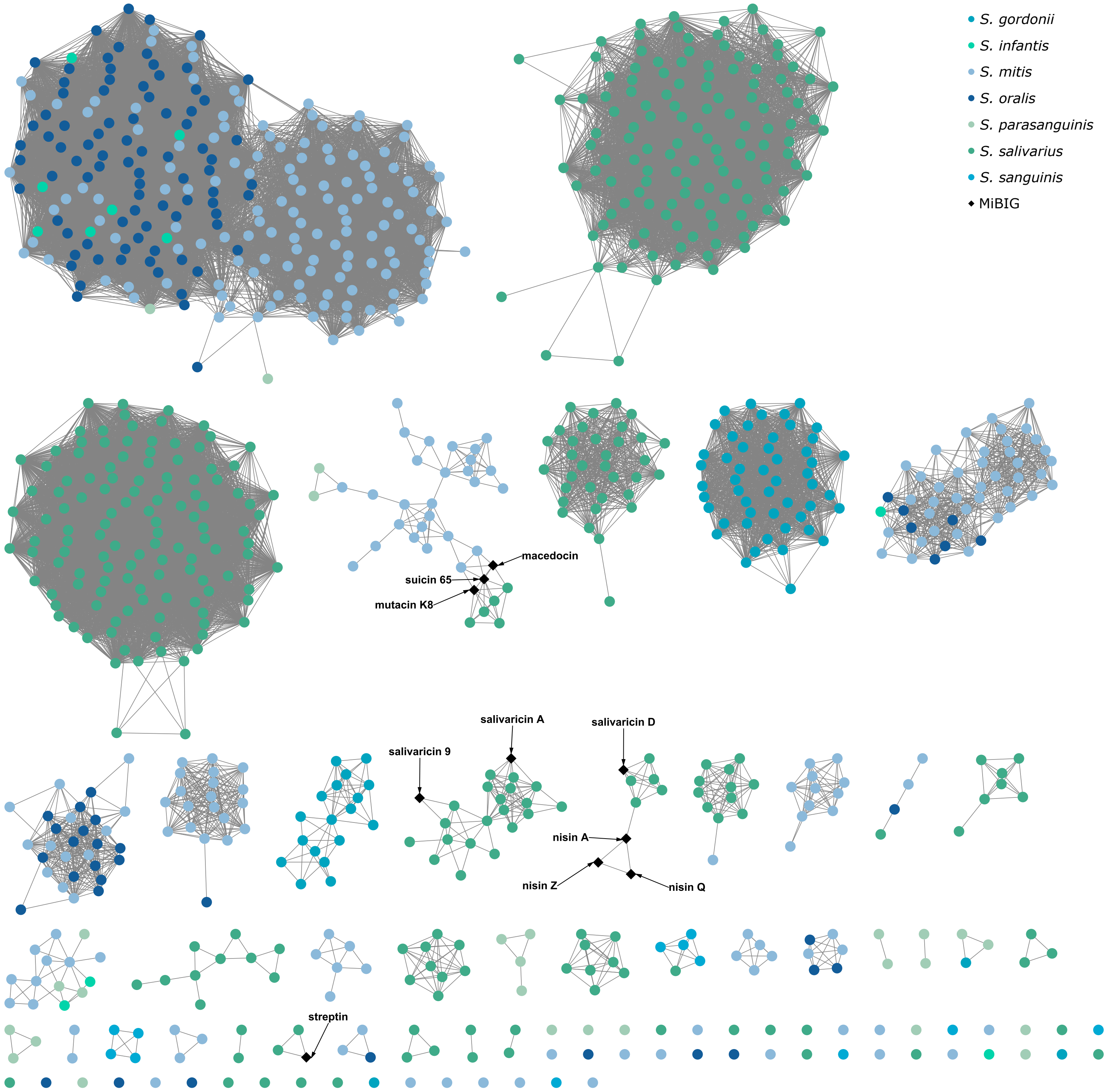

FIG S8 Identification of all RiPP-encoding BGC families from oral streptococci. In this network, each node represents a single RiPP-encoding BGC and edges indicate weighted pairwise distances between the corresponding BGCs. The nodes are colored based on the *Streptococcus* species as indicated by the key in the top right. Black diamonds represent characterized BGCs from the MIBiG database 1.4.

Table S1 Predicted BGCs from ADT and environmental bacterial genomes in the eHOMD genome database.

Table S2 Metagenome samples from the iHMP used in this study.
